# Positive selection and compensatory adaptation interact to stabilize non-transmissible plasmids

**DOI:** 10.1038/ncomms6208

**Published:** 2014-10-10

**Authors:** A. San Millan, R. Peña-Miller, M. Toll-Riera, Z. V. Halbert, A. R. McLean, B. S. Cooper, R. C. MacLean

**Affiliations:** 1 Department of Zoology, University of Oxford, Oxford OX1 3PS, UK; 2 Centro de Ciencias Genómicas, Universidad Nacional Autónoma de México, Cuernavaca 62210, México; 3 Centre for Tropical Medicine, Nuffield Department of Clinical Medicine, University of Oxford, Oxford OX3 7BN, UK; 4 Mahidol-Oxford Tropical Medicine Research Unit, Faculty of Tropical Medicine, Mahidol University, Bangkok 10400, Thailand

## Abstract

Plasmids are important drivers of bacterial evolution, but it is challenging to understand how plasmids persist over the long term because plasmid carriage is costly. Classical models predict that horizontal transfer is necessary for plasmid persistence, but recent work shows that almost half of plasmids are non-transmissible. Here we use a combination of mathematical modelling and experimental evolution to investigate how a costly, non-transmissible plasmid, pNUK73, can be maintained in populations of *Pseudomonas aeruginosa*. Compensatory adaptation increases plasmid stability by eliminating the cost of plasmid carriage. However, positive selection for plasmid-encoded antibiotic resistance is required to maintain the plasmid by offsetting reductions in plasmid frequency due to segregational loss. Crucially, we show that compensatory adaptation and positive selection reinforce each other’s effects. Our study provides a new understanding of how plasmids persist in bacterial populations, and it helps to explain why resistance can be maintained after antibiotic use is stopped.

## Abstract (web summary)

Plasmids are important for bacterial evolution but the evolutionary mechanisms behind their maintenance are unclear. Here the authors show that the interplay between compensatory adaptation and positive selection for plasmid-encoded antibiotic resistance determines plasmid persistence in *P. aeruginosa*.

## Graphical abstract

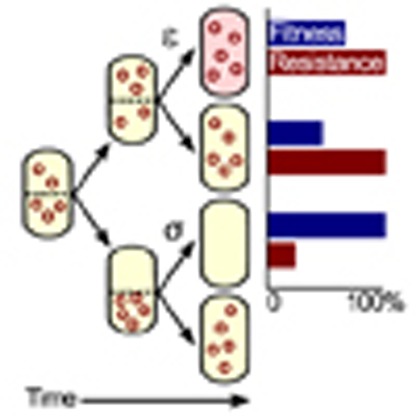

Plasmids are extra-chromosomal genetic elements coding for a wide range of traits that allow bacteria to adapt to different stresses and niches^1^. They are able to spread horizontally among bacteria by conjugation, and play a central role in the adaptation and evolution of prokaryotes^2^. For example, plasmids have played a huge role in the evolution of antibiotic resistance in bacterial populations^3^, which is currently one of the major threats to human health in developed countries^4^,^5^. Understanding the evolutionary pressures underlying the maintenance of plasmids in bacterial populations is therefore crucial to help prevent the menace of a post-antibiotic era.

Although plasmids play a pivotal role in bacterial evolution, it remains challenging to understand how plasmids can stably persist in bacterial populations^6^,^7^,^8^,^9^, and this is known as the plasmid paradox^10^. Two main factors are known to hinder plasmid maintenance: the burden produced by plasmids in the host cell and the rate of plasmid loss during cell division (segregational loss). Plasmids produce a multi-level fitness cost to host bacteria^11^. The origin of this cost comes from the burden imposed by the plasmid metabolism^12^,^13^, from specific regulatory interactions between plasmid and bacteria^14^,^15^, and from cytotoxic effects derived from the presence of the plasmid^11^. In addition to the cost they produce, plasmids can be lost during cell division, and despite the multiple mechanisms carried by plasmids to reduce the rate of segregational loss^16^,^17^, this rate remains strictly positive.

The key factors contributing to the maintenance of plasmids in bacterial populations are horizontal transfer, positive selection for plasmid-encoded genes and compensatory adaptation. Horizontal transfer, which usually occurs by conjugation, provides a simple mechanism for the spread of plasmids, both within and across species. If conjugation rates are sufficiently high, horizontal gene transfer can allow plasmids to persist in bacterial populations as parasites in spite of segregational loss and the cost of plasmid carriage. Nonetheless, the transfer conditions required for plasmids to act as pure genetic parasites are very restrictive and most of the previous studies^7^,^8^,^18^ (but not all^9^,^19^) reject this hypothesis based on theoretical considerations. Selection for plasmid-encoded genes provides a simple mechanism to maintain plasmids in bacterial populations over the short term. However, constant selection is thought to eventually lead to the incorporation of beneficial plasmid genes into the bacterial chromosome, rendering the plasmid redundant^8^,^20^. Finally, compensatory adaption by chromosomal and/or plasmid mutations alleviates the cost of plasmid carriage, reducing selection against it^10^,^20^,^21^,^22^,^23^,^24^. The general conclusion of classical mathematical models of plasmid population dynamics is that conjugation is necessary for the long-term survival of plasmids in bacterial populations, even if it is not itself sufficient to maintain plasmids^8^,^9^,^19^.

However, high-throughput analysis of plasmid sequences reveals that approximately half of known plasmids are not transmissible by conjugation (hereafter ‘non-transmissible’); that is, they are neither conjugative (able to self-transfer by conjugation) nor mobilizable (unable to self-transfer by conjugation but able to be transferred by a trans-acting conjugative element present in the same host bacteria)^25^,^26^,^27^. These analyses are based on the presence of genes involved in conjugation and mobilization on the plasmids, so they may underestimate the potential for the horizontal spread of plasmids by other means, such as transduction, natural transformation or co-integration into mobile genetic elements^25^. However, these alternative strategies for horizontal gene transfer are not expected to be as effective as conjugation^25^, and most non-transmissible plasmids probably rely largely on vertical transmission to be maintained in populations.

The widespread occurrence of non-transmissible plasmids challenges classical models of plasmid persistence, and highlights the need to develop new frameworks for thinking about plasmid evolution in the absence of horizontal transfer. In this paper we investigate how compensatory adaptation and rare episodes of positive selection for plasmid-encoded genes influence the persistence of non-transmissible plasmids at the population level. To address this problem, we combine an experimental model using the opportunistic pathogen *Pseudomonas aeruginosa* and the small non-transmissible plasmid pNUK73 (ref. 28) with mathematical modelling and whole-genome sequencing. We show that rare events of selection for plasmid-encoded traits interact with a rapid compensatory adaptation and are sufficient to maintain the plasmid in the population for hundreds of generations. This work contributes to our understanding of the general evolutionary pressures that maintain plasmids in bacterial populations, in particular providing a robust ecological and evolutionary mechanism that may explain the apparently paradoxical ubiquity of non-transmissible plasmids in natural populations.

## Results

### pNUK73 is rapidly lost in the absence of positive selection

We developed an experimental model system using *P. aeruginosa* strain PAO1 transformed with pNUK73, which is a small, multi-copy, non-transmissible plasmid carrying an aminoglycoside 3′-phosphotransferase, which confers resistance to kanamycin and neomycin^28^. The stability of non-transmissible plasmids in a bacterial population, in the absence of positive selection, depends on two factors: (i) the rate of segregational loss and (ii) the cost of plasmid carriage^29^. The rate of segregational loss will determine the frequency at which plasmid-free bacteria appear in the population. These cells will grow faster than plasmid-carrying bacteria, due to the cost produced by the plasmid, therefore increasing their frequency in the population over time.

The rate of segregational loss in small plasmids—such as pNUK73—that lack active partitioning and post-segregational killing systems depends on the number of units of partition of the plasmid in the bacterium. The units of partition are the plasmid entities available for segregation during cell division, which are determined by the plasmid copy number and the degree of plasmid multimerization^30^. pNUK73 exists mainly as a monomer (see methods), and the average copy per cell was 11.03 (s.e.=1.89, *n*=5). Assuming a zero variance in the plasmid copy number between cells and that plasmids are randomly distributed between daughter cells at cell division, we estimate that a lower bound for the rate of segregational loss of pNUK73 would be (0.5)^11^=4.79 × 10^−4^. To estimate the cost of plasmid carriage we directly competed PAO1/pNUK73 against a plasmid-free PAO1 strain carrying a neutral green fluorescent protein marker (GFP; ref. 27). pNUK73 produced a large fitness cost in PAO1; the relative fitness of PAO1/pNUK73 compared with PAO1 was 0.786 (s.e.=0.008, *n*=18).

**Figure 1:**
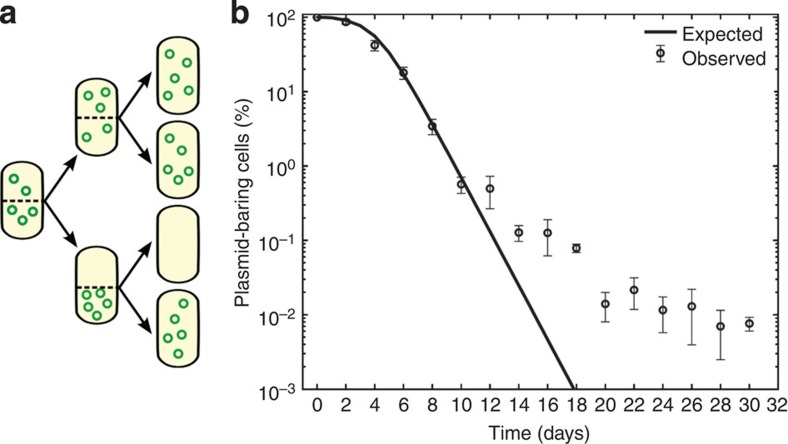
Stability of pNUK73 in the bacterial population. (**a**) Diagram illustrating the probability of a daughter cell not receiving any plasmid during division assuming no active partitioning system and random division of the plasmids (**b**) Relative proportion of plasmid-bearing bacteria (logarithmic scale) as a function of time. Expected (solid line) and observed (circles, average±s.d., *n*=3) dynamics of loss of plasmid pNUK73 in the population. Note how the numerical simulations of the model capture with quantitative accuracy the first 10 days of the experiments, but predict very different dynamics than observed experimentally after 10 days.

Mathematical models have previously been used to understand plasmid maintenance in the absence of selection, in particular to study the interaction between conjugation and segregational loss^6^,^8^,^9^,^31^,^32^, the effect of spatial structure^19^,^33^ and the effect of varying selection against plasmid carriage^24^,^34^,^35^. Theoretical results on the conditions that allow plasmids to persist in bacterial populations suggest that pNUK73 would be highly unstable because of the relatively low copy number (compared with other natural small plasmids^36^), the high fitness cost of this plasmid and the fact that it is non-transmissible. To predict the dynamics of pNUK73 carriage, we developed a simple mathematical model of plasmid dynamics in a bacterial population consisting of plasmid-bearing cells, and parameterized this model using data on the interaction between PAO1 and pNUK73 (Fig. 1 for model schematic, see methods for full model description). For the numerical simulations of the model, we will consider that the population is initially composed of entirely plasmid-bearing cells, and that plasmid-free cells are generated by segregational loss of the plasmid at a rate *σ*=4.79 × 10^−4^ per cell division (derived from the copy number of pNUK73). We assume that plasmid-bearing and plasmid-free cells compete for a limiting resource. A crucial feature of our model, which contrasts with the assumptions of previous models, is that it does not make any *a priori* assumptions about the fitness cost of plasmid carriage. Instead, we estimated resource uptake and conversion parameters for plasmid-bearing and plasmid-free cells based on the growth kinetics of pure cultures of PAO1 and PAO1/pNUK73. The fitness difference between these strains is therefore an emergent property of the model. In agreement with existing models of non-transmissible plasmid dynamics^34^,^35^,^37^, our model predicts that the frequency of pNUK73 should decrease exponentially as a result of both the large cost of plasmid carriage and the high rate of segregational loss (Fig. 1b). To test this prediction, we followed the dynamics of plasmid carriage in three populations of PAO1/pNUK73 that were propagated in antibiotic-free culture medium for 30 days, which corresponds to 300 generations of bacterial evolution (Fig. 1b). Over the first 10 days of the experiment, the frequency of plasmid-carrying cells declined exponentially, as predicted by our model and by previous theoretical models. Following this, the rate of decay in plasmid carriage slowed down, which does not agree with the theoretical predictions.

### Compensatory adaptation reduces the cost of pNUK73

**Figure 2:**
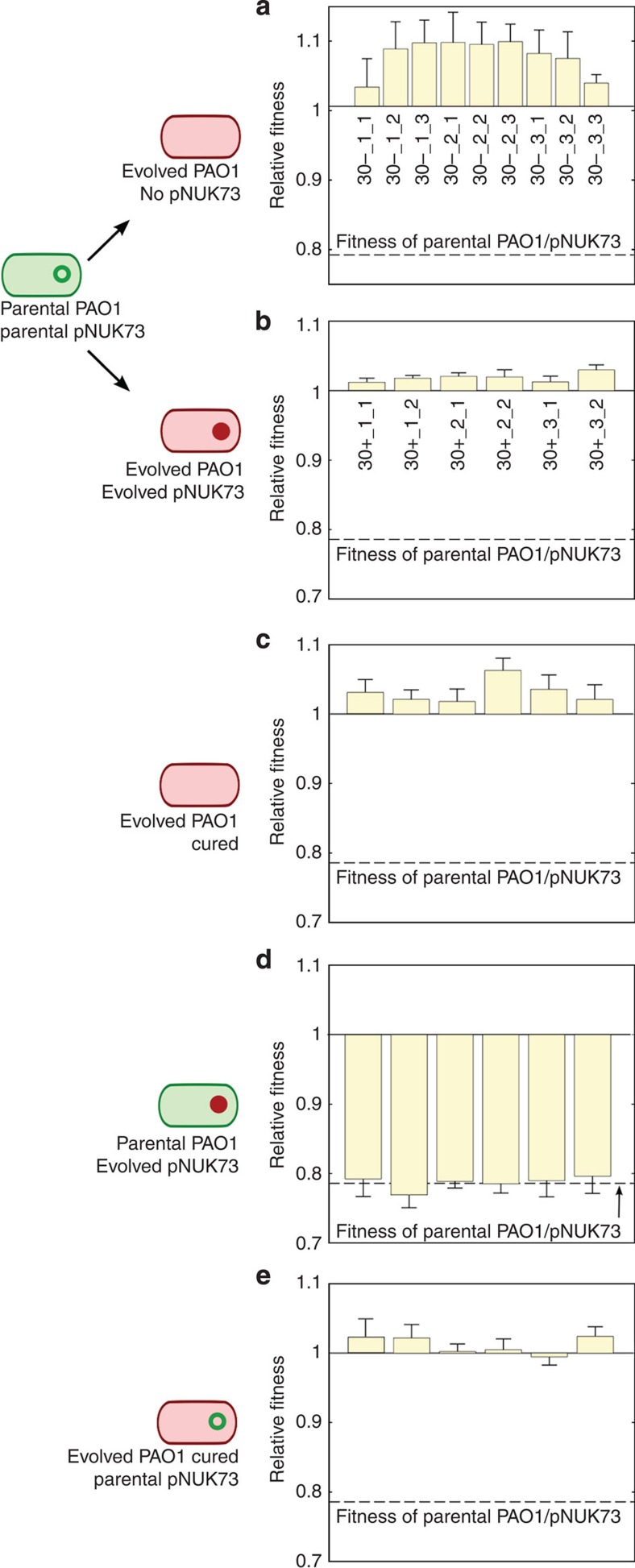
Compensatory adaptation to pNUK73 is due to changes in the host bacteria. Fitness (average±s.e.m., *n*=6), compared with the ancestral PAO1 of: (**a**) plasmid-free (three clones per population) and (**b**) plasmid-bearing clones (two clones per population) after 30 days of serial passage in the absence of antibiotics, (**c**) the cured clones from the evolved plasmid-bearing clones, (**d**) the parental PAO1 transformed with the six evolved pNUK73 plasmids and (**e**) the evolved cured clones transformed with the ancestral pNUK73. Note the small increase in fitness in the plasmid-free clones (**a**) and the compensation of the fitness cost produced by the plasmid (**b**) over the experiment. The plasmid produced no significant cost in the evolved clones (comparison between **b** and **c**) and the adaptation was due to changes in the bacterial host chromosome (comparison among the ancestral PAO1/pNUK73, **b** and **e**) and not in pNUK73 (comparison among the ancestral PAO1/pNUK73, **b** and **d**). The names of the clones are displayed in the *x*-axis and clones are arranged according to populations.

In the absence of positive selection, pNUK73 stability could increase as a result of a decrease in the rate of segregational loss, and/or a decrease in the cost of plasmid carriage. To test for a decrease in the rate of segregational loss, we assessed the copy number of pNUK73 in two plasmid-bearing clones isolated from each of the three populations at the end of the experiment. We found a slight but non-significant reduction in the plasmid copy number of the evolved populations (average=7.77, s.e.=0.79, *n*=3) compared with the ancestral population (11.03±1.89) (Two-sample *t*-test: *P*=0.171, *t*=1.59, df=5.21) (Supplementary Fig. 1). To test for a decrease in the cost of plasmid carriage, we competed plasmid-bearing clones (*n*=2 per population) and plasmid-free clones (*n*=3 per population) against the marked PAO1 reference strain (Fig. 2a,b). We estimated the effective cost of plasmid carriage by calculating the difference in mean fitness between plasmid-bearing (average=1.018, s.e.=0.002, *n*=3) and plasmid-free clones (average=1.073, s.e.=0.01, *n*=3). We found that plasmid-bearing clones were only at a small disadvantage compared with plasmid-free clones (6%) (two-sample *t*-test: *P*=0.025, *t*=−5.51, df=2.21), which was much lower than the substantial cost (21%) of plasmid carriage at the outset of the experiment (Fig. 2b,c). To determine the exact cost produced by pNUK73 at the end of the experiment, we cured the plasmid in the six PAO1/pNUK73 evolved clones by heat-shock^38^ and calculated their relative fitness. There was no difference in the fitness of the evolved clones (average=1.018, s.e.=0.002, *n*=3) and the evolved cured clones (average=1.032, s.e.=0.004, *n*=3, paired *t*-test, *P*=0.1544, *t*=−1.68, df=5), demonstrating that the plasmid no longer imposed a cost in the evolved clones (Fig. 2b,c). Thus, plasmid-bearing sub-populations had completely overcome the 21.4% cost that was initially associated with plasmid carriage, while plasmid-free sub-populations had only evolved an average 6% higher fitness.

These results showed that compensatory adaptation is able to fully overcome the cost produced by pNUK73; however, whether the modifications leading to the compensation were borne on the plasmid or in the bacterial chromosome remained unclear. To answer this question we transformed the ancestral PAO1 strain with the different evolved pNUK73 plasmids, and also transformed the different evolved and cured clones with the ancestral pNUK73 plasmid, and measured the fitness of these different lines relative to PAO1 (Fig. 2d,e). Clones of ancestral PAO1 carrying evolved pNUK73 showed the same low fitness (average=0.787, s.e.=0.004, *n*=3) as the ancestral PAO1/pNUK73 strain (two-sample *t*-test: *P*=0.9236, *t*=−0.10, df=21.5), while clones of evolved PAO1/pNUK73 cured and transformed with ancestral pNUK73 showed the same high fitness (average=1.012, s.e.=0.006, *n*=3) as the compensated PAO1/pNUK73 (paired *t*-test, *P*=0.308, *t*=1.13, df=5). These lines of evidence suggested that compensatory adaptation was due to chromosomal adaptation to the plasmid.

To understand the underlying genetic mechanisms driving adaptation in the evolved clones we used deep whole-genome sequencing (see methods) (Supplementary Table 1). As predicted by the fitness experiments, no mutations were found in the evolved plasmids. Every plasmid-bearing clone (*n*=6) carried a single chromosomal mutation in either a putative protein kinase (PA4673.15 or PA4673.16) or a putative helicase (PA1372) gene, suggesting that the bacteria had evolved compensatory adaptations that offset the cost of plasmid carriage. Plasmid-free clones (*n*=9) did not carry mutations in these genes mutated in plasmid-bearing clones, providing further evidence that kinase and helicase mutations are compensatory to the cost of the plasmid. Instead, seven out of nine plasmid-free clones carried mutations in the diguanylate cyclase *wspF* (PA3703) (two of the clones had an extra mutation in hypothetical genes); one of the remaining clones carried a mutation in a second diguanylate cyclase (PA5017) and one clone did not carry any mutations. Clones with *wspF* mutations had increased their fitness relative to the ancestral strain (average=1.076, s.e.=0.013, *n*=3), but *wspF* mutations did not compensate for the fitness cost associated with carrying pNUK73, as shown by the fact that transforming one of the *wspF* mutants with pNUK73 produced a similar fitness cost (20.3%) as in the ancestral PAO1 (21.4%, two-sample *t*-test: *P*=0.5582, *t*=0.64, df=3.6). These results indicated that mutations in the *wspF* gene represent an adaptation to the culture medium, conferring a similar small general advantage to both plasmid-carrying and plasmid-free clones, but do not compensate for the cost of pNUK73.

### Compensatory adaptation stabilizes pNUK73

**Figure 3:**
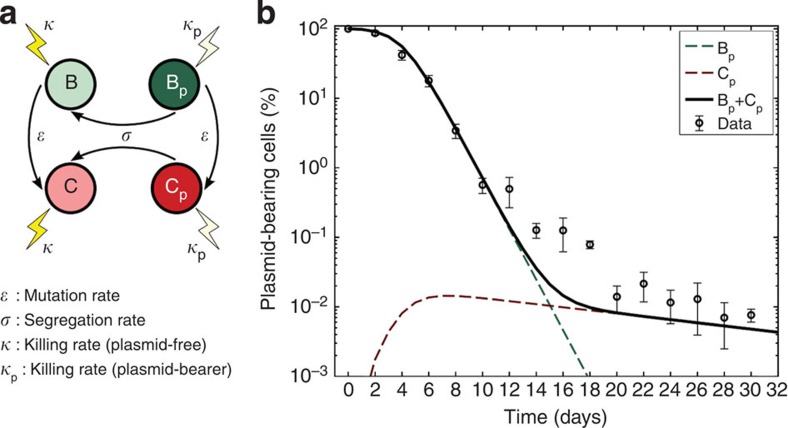
Compensatory adaptation stabilizes pNUK73 in the bacterial population. (**a**) Diagram illustrating the evolutionary dynamics of the model described in the methods section. (**b**) Relative proportion of plasmids-bearing bacteria (logarithmic scale) as a function of time. The solid black line represents the total expected frequency of plasmid bearers, while the red- and green-dotted lines denote the expected frequency of plasmid-bearing cells with and without the compensatory mutation. Circles represent the observed frequency of plasmid-bearing cells over time (average±s.d., *n*=3). Note that the predicted stabilization at the end of the experiment is a consequence of the compensated plasmid-bearing population having a higher fitness than the original parental strain and matches the experimental results.

To explore how compensatory adaptation impacts plasmid population dynamics, we extended our basic mathematical model so that populations consist of two bacterial genotypes, wild type (B) and compensated (C), that can either carry pNUK73 (B_P_ or C_P_) or not. We assume that compensatory mutations occur in both plasmid-bearing and plasmid-free populations at a rate *ε*, and we assume that compensatory mutations could not be reverted. As in our original model, we assume that the rates of segregational loss of plasmids in both the compensated and wild-type strains are determined by the plasmid copy number. Finally, we estimated resource uptake and conversion parameters for the compensated strain based on the growth kinetics of pure cultures of B, C, B_p_ and C_p_ clones. A schematic of the model is shown in Fig. 3a, with evolutionary dynamics given by equations (2a–f), 6, 7, 8, 9, 10 and parameters described in the methods section. Interestingly, including a compensated genotype into our model caused the frequency of plasmid bearers to stabilize after around 2 weeks, as was observed in the experiment (Fig. 3b). This effect emerges as a consequence of the increase in frequency of compensatory mutations in the plasmid-bearing sub-population. Because compensatory mutations reduce the cost of plasmid carriage, the rate of decline in the frequency of the plasmid declines as these mutations increase in frequency.

To experimentally confirm the effect of the specific compensatory mutations on the stability of pNUK73, we followed the dynamics of plasmid carriage in strains carrying a single compensatory mutation in either the helicase or the protein kinase for 10 days. As expected, the stability of pNUK73 after 10 days was much higher in the helicase (average=33.69%, s.e.=5.06%, *n*=6) and the kinase (average=32.40%, s.e.=4.66%, *n*=6) mutant populations than in the ancestral PAO1/pNUK73 population (average=0.57%, s.e.=0.08%, *n*=3) (Supplementary Fig. 2).

### Positive selection stabilizes pNUK73

Although compensatory adaptation decreases the rate at which non-conjugative plasmids are lost from populations, our model predicts that plasmids are still ultimately doomed to extinction (Fig. 3b). So, we ask, how can the plasmid be stabilized? Or, more precisely, based on previous theoretical results^37^, how will positive selection interact with compensatory adaptation to stabilize the plasmid? to address this question, we extended our model to include positive selection for plasmid-bearing cells by introducing a bactericidal antibiotic. To parameterize the model, we used a combination of minimal inhibitory concentration determination and growth curves in antibiotic-free medium to estimate the mortality rate of plasmid-bearing (*κ*_p_) and plasmid-free (*κ*) cells. We will also assume that the plasmid-bearing population degrades the antibiotic at a rate *α*_p_ and that the environmental antibiotic concentration decreases at a rate *α* as a consequence of antibiotic molecules binding to drug-susceptible cells.

**Figure 4:**
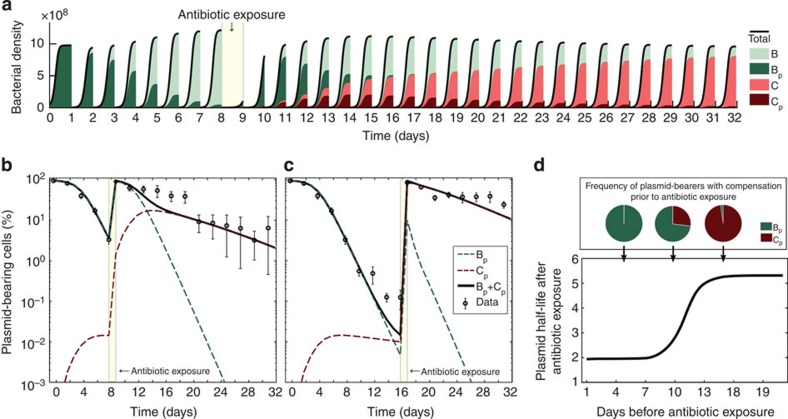
Compensatory adaptation and positive selection interact to stabilize the plasmid. (**a**) Simulated bacterial densities as a function of time in an experiment of duration 32 days, with drug deployed at day 8. The colour of each area denotes the frequency of each sub-population: plasmid-bearing parental strain in dark green, plasmid-free parental in light green, compensated plasmid-bearer in dark red and compensated plasmid-free in light red. (**b**,**c**) Relative proportion of plasmid-bearing bacteria (logarithmic scale) as a function of time. The solid black line represents the total expected frequency of plasmid bearers, while the red- and green-dotted lines denote the expected frequency of plasmid-bearing cells with and without the compensatory mutation. Circles represent the observed frequency of plasmid-bearing cells over time (average±s.d., *n*=3). Note how after antibiotic exposure at day 8 (**b**) or 16 (**c**) the plasmid-bearing population returns to 100% but with a different expected population structure than at the beginning of the experiment, and there is decay in the rate of plasmid loss in the population. (**d**) Expected plasmid half-life after antibiotic exposure as a function of the number of days elapsed before a single-day of antibiotic is used. If the drug is used early, then the population is composed mainly of the ancestral strain (illustrated in green in the pie chart above), while if there is a large delay before using the antibiotic then the plasmid becomes more stable because the population is now mostly composed of the compensated bacterial type (in red).

Figure 4a,b shows an example of our model, with a single-exposure to antibiotics at day 8. Of course, the use of the antibiotics has the effect of selecting for the plasmid-bearing population, thus resetting the frequency of plasmid bearers to a value near 100%. It is important to notice that, although at the beginning of the experiment and immediately after exposure to the antibiotic, the population is composed exclusively of plasmid-bearing cells, the genetic population structure in both these days is considerably different, and this has a substantial effect on the underlying stability of the plasmid. Indeed, a consequence of the genetic population structure changing over time is that the plasmid half-life following antibiotic exposure depends on the relative proportion of plasmid bearers with a compensatory mutation at the moment of antibiotic exposure. This can be explained as follows. When antibiotic exposure is removed, plasmid frequency always declines, but the rate at which the plasmid declines in frequency depends critically on the genetic structure of the bacterial populations prior to exposure to the antibiotic. If the frequency of compensatory mutations in the plasmid-bearing sub-population is low prior to exposure, then the frequency of the plasmid will decline rapidly following antibiotic exposure as a result of both the cost of resistance and segregational loss. In contrast, if all plasmid-bearing cells carry compensatory mutations prior to antibiotic exposure, then the frequency of the plasmid will decrease slowly after exposure. Because the frequency of compensatory mutations in the plasmid-bearing sub-population must increase through time, our model predicts that the plasmid becomes more stable as the number of days before antibiotic exposure increases, as illustrated in Fig. 4b–d. In summary, the mathematical model presented in this section suggests that compensatory mutations can make the plasmid more stable, and that rare events of selection are sufficient to maintain pNUK73 in the population. Furthermore, the model predicts that the stability of the plasmid depends on the genetic population structure, and as a result the plasmid becomes more stable every time we positively select for it.

**Figure 5:**
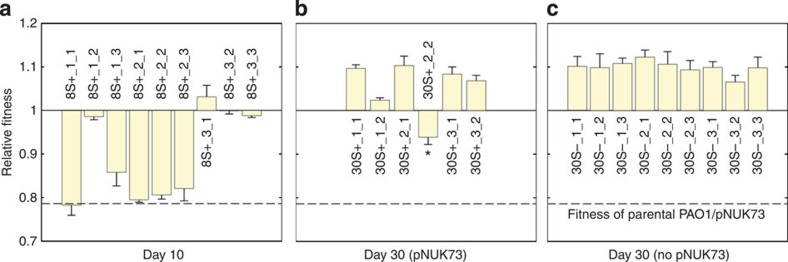
Relative fitness of clones from populations treated with neomycin. Fitness (average±s.e.m., *n*=6), compared with the ancestral PAO1, of (**a**) nine plasmid-bearing clones (three per population) after one step of selection with neomycin from day 8 to 9, and (**b**) six plasmid-bearing clones (two per population) and (**c**) nine plasmid-free clones (three per population) from the same populations at day 30. Note that, as predicted by the model, immediately after antibiotic exposure (day 10) the population is composed both of PAO1/pNUK73 clones with the compensation and with a relative fitness similar to that of the parental. At the end of the experiment, however, all the plasmid-bearing clones have had a fitness cost compensatory mutation. Note that one of the plasmid-bearing clones at day 30 is marked with a star (30S+_2_2). This clone was excluded from the statistical analyses due to discrepancies between the fitness measurements in tubes and 96-well plates. The names of the clones are displayed in the *x*-axis and clones are arranged according to populations.

To validate the predictions from the mathematical model we experimentally tested the effect of positive selection on the stability of pNUK73. We applied one step of overnight selection using neomycin after day 8 or 16 and then we monitored the frequency of the plasmid until day 30 (Fig. 4b,c). As predicted by the model, the rate of decay in the frequency of plasmid-bearing cells became much lower after the antibiotic treatment compared with the ancestral populations. To test the prediction that an increase in the stability of plasmid following positive selection is due to an increase in the frequency of compensatory mutations in the plasmid-bearing population, we tested for compensatory adaptation in nine clones (three per population) that were isolated immediately following treatment with neomycin at day 8 (Fig. 5a). We found that four out of nine clones had compensated the cost of plasmid carriage (fitness between 0.986 and 1.032) and whole-genome sequencing identified mutations again in the putative helicase (PA1372) and in one of the putative protein kinases (PA4673.16) in these four clones, demonstrating that increased fitness was due to compensatory adaptation (Supplementary Table 1). The five remaining clones lacked mutations in the putative helicase (PA1372) and putative protein kinases (PA4673.15-16) and presented a relative fitness similar to the ancestral PAO1/pNUK73 (fitness between 0.783 and 0.858) (Fig. 5a, Supplementary Table 1). These results support the prediction that compensatory adaptation increases plasmid stability following positive selection. Alternatively, it is possible that the stability of the plasmid increases following treatment as a result of increased copy number. However, we found that the plasmid copy number of clones (two per population) isolated after treatment (average=9.56, s.e.=1.22, *n*=3) was not significantly different from the copy number of pNUK73 in the ancestral strain (11.03±1.89) (two-sample *t*-test: *P*=0.537, *t*=0.65, df=5.96).

### Antibiotic accelerates general adaptation of PAO1/pNUK73

**Figure 6:**
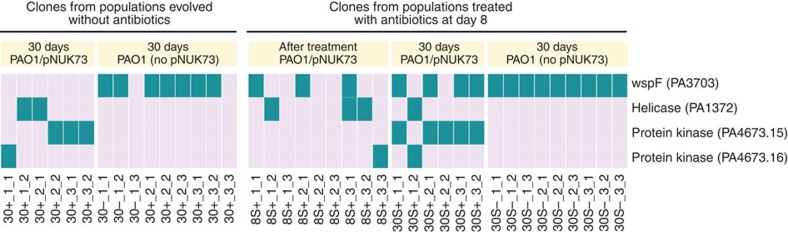
Common mutations in the clones of this study. This figure shows the distribution of mutations in the four most commonly targeted genes in this study across the clones that we assayed. Note that mutations in the putative helicase (PA1372) and the two putative kinases (PA4673.15-16) are only present in plasmid-bearing clones and are responsible for the compensation for the plasmid cost. The names of the clones are displayed in the *x*-axis and clones are arranged according to populations.

The evolution of increased fitness due to *wspF* mutations in plasmid-free lineages highlights the fact that it was possible for bacteria to acquire general adaptations that increased fitness independent of plasmid carriage over the course of our selection experiment. Theoretical considerations suggest that the frequency of positive selection for plasmids mediated by antibiotic exposure should play a key role in the general adaptation of plasmid-bearing lineages. By increasing the size of the plasmid-bearing sub-populations, positive selection for plasmid-bearing clones should accelerate the rate of production of general beneficial mutations, resulting in an increased rate of general adaptation. Consistent with this idea, we found that fitness at the end of the experiment (day 30) was higher in plasmid-bearing clones from populations that were treated with antibiotics at day 8 (Fig. 5b, mean fitness=1.080, s.e.=0.013, *n*=3) than in untreated control populations (Fig. 2b, mean fitness=1.018, s.e.=0.002, *n*=3), as judged by a two-sample *t*-test (*P*=0.0359, *t*=4.81, df=2.12). This difference in fitness cannot be explained by compensatory adaptation, as whole-genome sequencing demonstrated that all plasmid-bearing clones carried a compensatory mutation (Fig. 6, Supplementary Table 1). However, every clone from treated populations carried a second mutation, whereas none of the clones from untreated populations carried any other mutations (Fig. 6). Four out of six of the extra mutations found in treated clones were beneficial *wspF* mutations and we speculate that the mutations carried by the remaining two clones are alternative general beneficial mutations (Supplementary Table 1).

An important distinction exists between the absolute cost of plasmid carriage, which is expressed in terms of the reduction in fitness associated with carrying the plasmid for an individual clone, and the effective cost of plasmid carriage, which is measured in terms of the mean difference in fitness between plasmid-bearing and plasmid-free clones from the same population. In the untreated control populations, selection for compensatory adaptation was able to eliminate the absolute cost of plasmid carriage, but plasmid carriage was still associated with a 6% effective cost because plasmid-bearing lineages did not evolve general adaptations to the culture medium due to their small population size. In contrast, the increased rate of adaptation in plasmid-bearing lineages from populations that were treated with antibiotics at day 8 was able to eliminate the effective cost of plasmid carriage (Fig. 5b,c) as shown by a two-sample *t*-test between plasmid-bearing (mean fitness=1.080, s.e.=0.013) and plasmid-free (mean fitness=1.099, s.e.=0.006) clones at the end of the experiment (*P*=0.2624, *t*=−1.39, df=2.88).

## Discussion

Non-transmissible plasmids are common in both pathogenic and environmental bacteria^25^,^27^,^39^,^40^,^41^ and it is difficult to understand how these plasmids can persist in bacterial populations as a result of the cost of plasmid carriage. To understand the ecological and genetic mechanisms that promote the stability of plasmids, we deliberately chose to work with a plasmid (pNUK73) that is highly unstable in *Pseudomonas* populations due to the fact that it has a high rate of segregational loss and imposes a substantial cost. Classical models of plasmid evolution predict that conjugation is necessary for plasmid maintenance^6^,^7^,^8^,^9^, but we found that compensatory adaptation to ameliorate the cost of plasmid carriage coupled to rare selection for plasmid-encoded antibiotic resistance was sufficient to stabilize this bacteria/plasmid association. We therefore argue that our study presents a new understanding of why non-conjugative plasmids are so common, and also helps to explain why resistance genes persist in bacterial populations even in the absence of antibiotic use.

At the start of our experiment, the frequency of the pNUK73 plasmid rapidly declined, but the rate of decline in plasmid frequency slowed down as a result of chromosomal compensatory adaptation that removed the cost of plasmid carriage (similar kinetics of decline in antibiotic resistance have been observed in natural populations)^42^. However, compensatory adaptation was not sufficient to stably maintain pNUK73. First, although compensatory adaptation eliminated the absolute cost of plasmid carriage, segregational loss of plasmids continually decreased plasmid frequency. Second, relatively common plasmid-free lineages acquired more general beneficial mutations, compared with relatively rare plasmid-bearing lineages, resulting in an effective cost of plasmid carriage. Crucially, we found that selection for plasmid-encoded antibiotic resistance was necessary to maintain the plasmid, as has been observed before^43^. The immediate effect of antibiotic exposure was that the frequency of the plasmid increased to almost one. Following this immediate ‘rescue’ effect, the frequency of the plasmid declined, but the rate of decline following treatment gradually slowed as compensatory mutations spread through the plasmid-bearing population. In other words, compensatory adaptation resulted in an increased plasmid half-life following antibiotic exposure (Supplementary Fig. 3). The long-term consequence of antibiotic exposure was that increased population size allowed plasmid-bearing lineages to evolve general adaptations, effectively eliminating selection against plasmid carriage. Therefore, we argue that it is the interaction between compensatory adaptation and positive selection that helps to stabilize non-conjugative plasmids in bacterial populations.

As a result of the large cost imposed by pNUK73, there was strong selection to minimize the cost of plasmid carriage. The ability of bacteria to evolve compensatory mutations that offset the cost of pNUK73 played an important role in stabilizing the plasmid. A number of studies have shown that compensatory adaptation rapidly eliminates the cost of plasmid carriage^21^,^23^,^44^,^45^, but the precise genetic mechanisms underpinning adaptation to the cost of plasmid carriage remain poorly characterized. We found that mutations in three chromosomal genes completely compensated the cost of the plasmid without imposing any additional fitness burden (Fig. 2b,c). The compensatory mutations that we identified are found in genes that code for a putative helicase carrying an UvrD-like helicase C-terminal domain, and two contiguous putative serine/threonine protein kinases (Fig. 6). The specific role of these mutations in the molecular mechanisms of compensation will be investigated explicitly in a future study.

Although it is clear that compensatory adaptation and positive selection can maintain non-conjugative plasmids on a time scale of hundreds of generations, the maintenance of these plasmids over longer time scales remains enigmatic, because plasmid-encoded genes could theoretically move to the chromosome, rendering the plasmid redundant. In this case, we would expect that the plasmid would gradually be eliminated from the population due to segregational loss, even if the plasmid carried no cost. One potential resolution to this dilemma is that some genes carried on non-conjugative plasmids may confer a greater fitness benefit when they are carried on plasmids than when they are integrated into the chromosome, for example, because carrying multiple copies of the gene enables high levels of expression, or because heterogeneity in plasmid copy number generates adaptive heterogeneity in gene expression between plasmid-bearing cells. Second, it is possible that rare episodes of horizontal transfer might allow the maintenance of non-conjugative plasmids. Mechanisms such as transduction or co-integration with other mobile genetic elements that could facilitate the horizontal transfer of non-conjugative plasmids probably play an important role. In the future, we will extend the approach developed here to understand the mechanisms that permit the long-term maintenance of non-transmissible plasmids.

## Methods

### Plasmid dynamics model

Let us assume that our bacterial population consists of the following phenotypes: parental strain, both plasmid free (*B*) and plasmid bearing (*B*_p_), as well as a compensated bacterial genotype, again both plasmid free (*C*) and plasmid bearing (*C*_p_). For the purpose of this paper we will consider that the parental genotype can acquire a compensatory adaptation through a single-point mutation occurring with a probability *ε*>0, both in the plasmid-free and plasmid-bearing populations. For simplicity we will consider that reversion from compensation to wild type never occurs.

We will also assume that the bacterial population grows in a homogeneous environment and under resource limitation. So, if we denote the environmental concentration of the limiting resource at time *t* with the variable *R*(*t*), then we can model the per-cell uptake of the resource that corresponds to each phenotype with a standard Monod function of the environmental concentration of the resource,




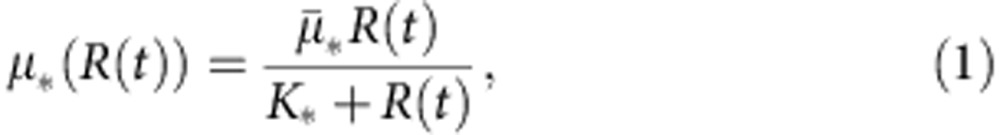




where * represents a placeholder for the corresponding bacterial type, 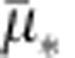 the maximum uptake rate and K_*_ the half-saturation constant. Then growth rate of bacterial type * can be described as a product of a resource conversion rate, denoted by ρ_*_, multiplied by the resource uptake equation (1), an expression that can be written as G_*_(*R*)=ρ_*_μ_*_(*R*). Note that the fitness cost associated with carrying a plasmid can be expressed by the plasmid-bearing population presenting a lower growth rate than the plasmid-free population G_*p_(*R*)≤G_*_(*R*).

The small plasmid considered in this paper lacks an active partitioning system or a post-segregational killing mechanism. Therefore we can model the rate of segregational loss based on the units of partition in the bacterium, a property determined by the plasmid copy number and the degree of plasmid multimerization. In particular, as the plasmid is present in the cell as a monomer, then each plasmid copy can be considered as a unit of partition and therefore the rate of segregational loss can be estimated by considering a random spatial distribution of the different copies of the plasmid at the moment of cell division. Indeed, the probability of a given copy of the plasmid not entering a specific daughter cell is 0.5 and, therefore, if we consider there are *n* plasmids in the dividing cell, the probability that none of these plasmids will enter a specific daughter cell is (0.5)^*n*^. Consequently, the probability that one of the daughter cells receives no plasmids can be approximated by a binomial distribution from which we can derive a rate of segregational loss, hereafter denoted by *σ*.

Also, we will denote the concentration of antibiotic in the environment with the variable *A*(*t*). Because the experimental model system under consideration is based on a well-mixed environment, we can assume mass action kinetics between antibiotic molecules and bacterial cells, and therefore we will model the bactericidal effect of the antibiotic by considering it kills susceptible cells at a rate *κ*. As plasmid-bearing cells present higher levels of antibiotic resistance, then the killing rate of plasmid-bearing cells by the antibiotic, *κ*_p_, satisfies the inequality 0≤*κ*_p_<*κ*<1.

The resistance mechanism we are modelling is based on the inactivation of antibiotic molecules by a drug-degrading enzyme, so we will consider that the rate of antibiotic reduction in the environment as a consequence of the plasmid-bearing population is given by *α*_p_. Similarly, *α* will represent the rate of decrease in drug concentration due the action of the antibiotic on the susceptible cells.

Our experimental setup consists of a batch protocol with *N* serial dilutions, performed every *T*=24 h. If we denote each transfer with the superscript *i*={0,1,2, …,*N*}, then the state of the system on day *i* can be represented with the vector **x**^*i*^(*t*)=(*A*^*i*^(*t*), *R*^*i*^(*t*), *B*^*i*^(*t*), *B*_p_^*i*^(*t*), *C*^*i*^(*t*), *C*_p_^*i*^(*t*)). In our experimental protocol we can decide at the beginning of each day if we can use drug or not, that is *A*^*i*^(0)=*A*_0_ or *A*^*i*^(0)=0, where *A*_0_ represents a lethal concentration of antibiotic.

Furthermore, we will consider, both in the numerical simulations (performed using a standard Matlab Ordinary Differential Equations solver) and in the experiments presented in this paper, that the initial inoculum is composed exclusively of parental, plasmid-bearing bacteria (at a density denoted by *B*_0_). Therefore the initial conditions of the first day can be written as 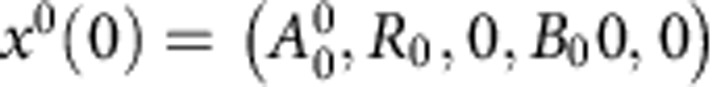. The following days the initial density of each bacterial phenotype will be determined as a projection of the terminal condition of the previous day, with fresh medium and antibiotic. Therefore the initial condition of transfer *i*>0 will satisfy the following condition:




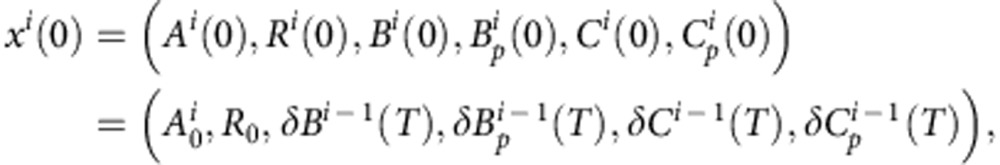




where 0<*δ*≪1 represents the dilution parameter. Then the evolutionary equations that describe the dynamics of each day can be written as:




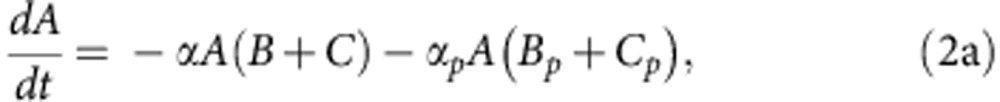







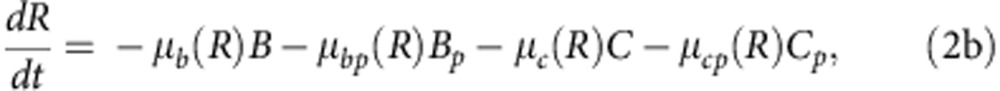







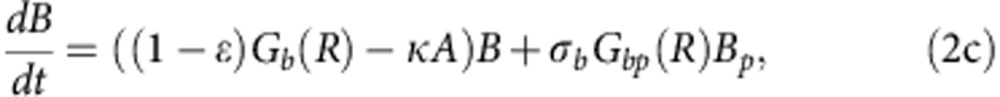







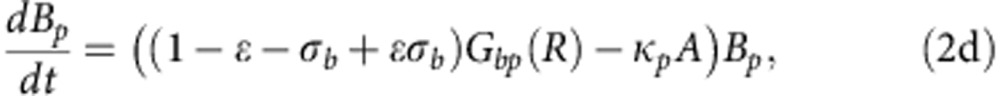







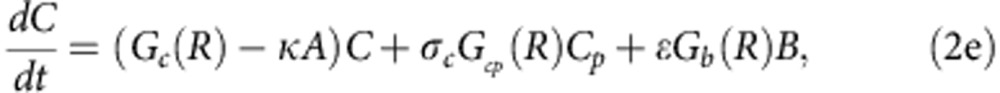







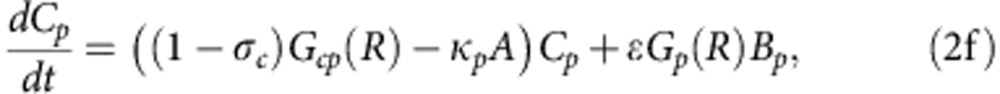




suitably augmented with initial conditions.

### Parameter determination

The benefit of using a resource-based modelling approach like the one presented in this paper, is that we can determine the growth kinetics and antibiotic suppression parameters for different bacterial types independently, with the aim of predicting the ecological and evolutionary dynamics of competition experiments. In particular, the parameterization process used in this paper uses a Metropolis–Hastings Markov-chain Monte Carlo method described in the Supplementary Information (Supplementary Methods, Supplementary Figs 5–9, and Supplementary Tables 2 and 3). Identifiability of the growth kinetic parameters was confirmed using a data cloning method^46^,^47^. Also, we used dose–response experiments to quantify the susceptibility patterns of each phenotype to calibrate the parameter used to model the effect of the antibiotic on each bacterial type (see Supplementary Fig. 4). Segregation rate for the parental and evolved strains was determined from the plasmid copy number, a quantity measured using quantitative PCR (qPCR) and validated by the whole-genome sequence analysis. The resulting best-fit parameters used in the numerical simulations of the model are summarized in Table 1.

### Bacterial strains, plasmids and culture conditions

*P. aeruginosa* PAO1 was electroporated with plasmid pNUK73 as previously described^48^. Plasmid pNUK73 does not belong to any known incompatibility group, and only carries a replication gene, an aminoglycoside 3′-phosphotransferase gene conferring resistance to kanamycin and neomycin and a *lacZ* gene (GenBank accession no. AB084167). The dynamics of plasmid carriage in PAO1/pNUK73 populations was followed every 2 days by plating on LB agar plates (Fisher Scientific, NJ, USA) and LB agar plates containing 50 mg l^−1^ of neomycin (Sigma Chemical Co., St. Louis, MO, USA) to estimate the total number of bacteria and bacteria containing pNUK73, respectively. Aliquots of serial dilutions (10^−2^–10^−6^) were plated in duplicate and plates containing 30–300 colonies were used for the analysis (average of two plates). The presence of pNUK73 in the colonies growing on LB+neomycin was confirmed by PCR using primers specific for the putative origin of replication of pNUK73 (forward: 5′-CGCTAAGGATGTTTACAC-3′, reverse: 5′-CTCAACCGTTCTAGGATT-3′) in a subset of this colonies (10 per time point) using GoTaq green Mastermix (Promega, Madison, WI, USA). Minimal inhibitory concentrations of neomycin were performed in triplicate following Clinical and Laboratory Standards Institute guidelines^49^. Growth curves were done using a BioTek Synergy H4 plate reader (BioTek Instruments, Potton, UK) using 96-well plates; overnight cultures of the different clones were diluted 1:100 in LB broth and OD_600_ was measured every 20 min for 18 h at 37 °C. The degree of multimerization of plasmid pNUK73 in PAO1 was determined as in the study by Summers and Sherratt^30^ using plasmid obtained using both the Birnboim and Doly method^50^ and QIAprep Spin Miniprep (Qiagen, Inc., Chatworth, CA, USA). Plasmid curing was performed subjecting overnight bacteria cultures to heat-shock by four cycles of incubation at different temperatures; 0 °C for 30 min and then 37 °C for 30 min (ref. 38), aliquots of the cultures were then plated on LB agar and 100 colonies were replicated on LB agar and LB agar containing 50 mg l^−1^ of neomycin. The clones growing on LB agar but not on LB+neomycin were confirmed as cured from the plasmid by a negative pNUK73-specific PCR (as described above).

The *wspF* mutant strain used to assess the cost of pNUK73 in the *wspF* mutant background was 30-_1_1. Clones 30+_1_2 (putative helicase mutant) and 30+_2_2 (putative protein kinase mutant) were used to check pNUK73 stability in plasmid-compensated mutants (six populations per clone). See Supplementary Table 1 for clone nomenclature.

### Quantification of pNUK73 copy number by qPCR

The copy number of pNUK73 was determined by qPCR using an ABI StepOnePlus Real-Time PCR System (Life Technologies, USA). DNA extraction, quantification and digestion were performed as previously described^27^. We developed a specific qPCR for pNUK73 (pNUK73-forward: 5′-AGTGTAAAGCCTGGGGTGC-3′, pNUK73-reverse: 5′-GTCTGCGTTGTCGGGAAGA-3′, size: 199 bp, efficiency: 93.22%) and we used a previously described qPCR of the *rpoD* chromosomal monocopy gene (rpoD-forward: 5′-GGGCGAAGAAGGAAATGGTC-3′, rpoD-reverse: 5′-CAGGTGGCGTAGGTGGAGAA-3′, size: 178 bp, efficiency: 98.51%)^51^ to compare the ratio of plasmid and chromosomal DNA. Efficiency of the reactions was calculated from the standard curve generated by performing qPCR with four 10-fold dilutions of template DNAs in triplicate (~2 ng μl^−1^ to 2 pg μl^−1^ working range of DNA concentration). qPCRs were performed using ABI SYBR Select Master Mix (Life Technologies) at a final DNA concentration of 0.2 ng μl^−1^. The amplification conditions were: initial denaturation for 10 min at 95 °C, followed by 30 cycles of denaturation for 15 s at 95 °C, annealing and extension for 1 min at 60 °C. After the amplification was complete, a melting curve analysis was performed by cooling the reaction to 60 °C and then heating slowly to 95 °C. Inter-run calibration samples were used to normalize the results from different plates of each qPCR.

To calculate the copy number of plasmid per chromosome we used the formula:




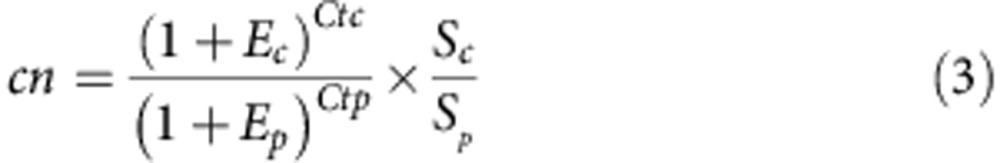




where cn is the plasmid copy number per chromosome, *S*_c_ and *S*_p_ are the sizes of the chromosomal and pNUK73 amplicons (in bp), *E*_c_ and *E*_p_ are the efficiencies of the chromosomal and plasmid qPCRs (relative to one), and Ctc and Ctp are the threshold cycles of the chromosomal and plasmid reactions, respectively.

### Competitive fitness assays

The fitness of each clone was determined relative to the PAO1–GFP strain as described in San Millan *et al.*^27^ Six biological replicates of the competition were performed for each clone. Pre-cultures of the strains were incubated at 37 °C with 225 r.p.m. shaking overnight in 3 ml of LB broth. Pre-cultures were diluted 20-fold in 200 μl of fresh LB and incubated in the same conditions in 96-well plates until they reach mid-exponential phase (OD_600_≈0.5). Cultures of the strains were then mixed at a ratio of ~50% clone under study to 50% PAO1–GFP. The exact initial proportions were confirmed via flow cytometry using an Accuri C6 Flow Cytometer Instrument (BD Accuri, San Jose, CA, USA). Mixtures were diluted 400-fold in fresh LB and competed for 16 h at 37 °C with 225 r.p.m. shaking (~eight generations). Again, the final proportion was measured by flow cytometry. The fitness of the strain carrying the plasmid(s) relative to the PAO1–GFP strain was determined using the formula:




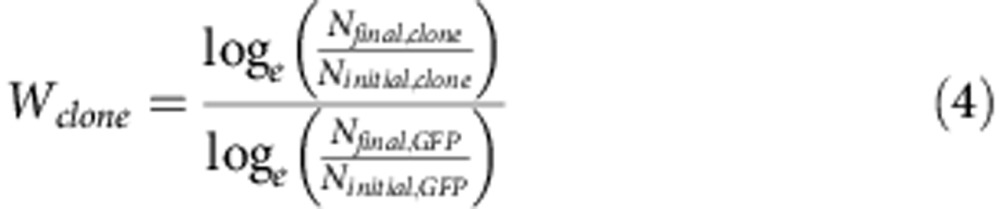




where *W*_*clone*_ is the fitness of the PAO1 clone under study, *N*_*initial,clone*_ and *N*_*final,clone*_ are the numbers of cells of the strain carrying the plasmid before and after the competition, and *N*_*initial,GFP*_ and *N*_*final,GFP*_ are the numbers of cells of PAO1–GFP before and after the competition. As a control PAO1–GFP was competed against an unmarked PA01 strain in every competition, the average fitness of PAO1–GFP relative to PAO1 was 0.993 (s.e.=0.012, *n*=36).

Competitions experiments were done in 96-well plates instead of in tubes (as the experimental evolution) to increase the throughput of the experiments. Although both the tubes and the plates were incubated at 225 r.p.m., the effective shaking is much higher in the tubes. As a control, a subset of clones (10) with a wide range of relative fitness (0.79–1.08) and including mutants in the most common targeted genes (PA3703, PA1372 and PA4673.15-16) was competed against PAO1–GFP both in tubes and 96-well plates and the results were consistent in both conditions (*R*^2^=0.94). Only one clone in the experiment, 30S+_2_2, carried a mutation in a flagellar gene (Supplementary Table 1). Alterations in the flagellum have been related to variations in fitness depending on the shaking conditions of the cultures^52^,^53^. Therefore we measured the relative fitness of 30S+_2_2 in both conditions. This clone was the only one analyzed showing a big difference in relative fitness in the tubes (*w*=1.098, s.e.=0.005) and in the 96-well plates (*w*=0.939, s.e.=0.040). Therefore we excluded this clone from the statistical analyses for relative fitness.

One potential problem for the estimation of fitness in the plasmid-carrying clones would be if the compensatory mutations altered the rate of segregational loss of pNUK73, leading to differences in plasmid stability during the competitions. To test for this possibility the percentage of plasmid-bearing cells was tested in the PAO1/pNUK73 as well as in two compensated mutants (30+_1_2 helicase mutation and 30+_3_1 kinase mutation) after culturing them using the exact same conditions as in the actual competition experiments. Stability was measured as explained above in three independent populations of each clone. The differences in stability among the clones were not significant (PAO1/pNUK73: average=85%, s.d.=7.7%; 30+_1_2: average=96.6%, s.d.=4.6%; 30+_3_1: average=92.9%, s.d.=9.5.; analysis of variance: *P*=0.269, *F*=1.443, df=1, 7). Therefore, the differences of plasmid stability in the different clones should not affect the results of the fitness determinations.

### Statistical analyses

Analyses were performed using R (version 2.14.1). Two plasmid-bearing clones and three plasmid-free clones were analyzed for each population, both for relative fitness and plasmid copy number (when applicable). The different clones within a population could not be considered independent; therefore the values for the clones within each population were averaged for the analyses. Comparisons among evolved clones, cured clones and re-transformed clones were done using paired *t*-tests and all the other comparisons were done using two-sample *t*-tests.

### Whole-genome sequencing and bioinformatics

DNA extractions were performed from 2 ml of LB broth cultures incubated at 37 °C with 225 r.p.m. shaking overnight using the Qiagen DNeasy Blood and Tissue Kit (Qiagen, Inc.). DNA was quantified using the QuantiFluor dsDNA system (Promega), following manufacturers’ instructions. All sequencing was conducted at the Wellcome Trust Centre for Human Genetics using HiSeq2000 and 100-bp-paired-end reads. Raw reads were initially filtered using the NGS QC Toolkit^54^. First, reads were trimmed from the 5′ and/or 3′ end if the PHRED quality score was <20. After the trimming step, reads shorter than 50 bp were eliminated. Reads were also discarded if >2% of their bases were ambiguous or if <80% of their bases had a PHRED quality score <20. The ancestral strain used in this study had two additional features regarding the reference genome *P. aeruginosa* PAO1 (NC_002516.2): plasmid pNUK73 (AB084167.1) and the insertion of the phage RGP42 (GQ141978.1). Filtered reads were mapped to the *P. aeruginosa* PAO1 reference genome, the plasmid pNUK73 and the phage RGP42 using Burrows-Wheeler transform (BWA)^55^. The average median depth of coverage across clones was 60 × . Mapped reads were subsequently processed to diminish the occurrence of false positives in further downstream analyses: (1) reads with multiple best hits were discarded; (2) duplicated reads were eliminated using the MarkDuplicates tool from the Picard package ( http://picard.sourceforge.net); (3) reads were locally realigned around indels (these regions are prone to misalignment) using RealignerTargetCreator and IndelRealigner from the GATK package^56^; and (4) reads mate information was fixed and sorted using the FixMateInformation command in the Picard package ( http://picard.sourceforge.net).

Variant calling was performed using two different approaches: Samtools’s mpileup^55^ and GATK’s Unified Genotyper^56^. The raw variants were filtered for strand bias, end distance bias, base quality bias, single nucleotide polymorphisms (SNPs) around gaps, low coverage and erroneously high coverage using VCFtools (vcf-annotate)^57^ and GATK toolkit (VariantFiltration)^56^. High quality variants were subsequently annotated using SnpEff^58^. Structural variants were detected using three different approaches. First, Breakdancer^59^ was used to predict five types of structural variants—deletions, insertions, inversions, inter- and intra-chromosomal translocations—using information from read pair mapping. The output of Breakdancer was passed to Pindel^60^, which can use calls from Breakdancer to increase its performance. Pindel infers deletions, short insertions, long insertions, inversions, tandem duplications and breakpoints using a split-read approach. Finally, to detect copy number variants Control-FREEC^61^ was used. Control-FREEC identifies copy number variants using depth of coverage and normalization by GC content. Regions with low mappability can be excluded from the analysis by providing Control-FREEC with mappability tracks. Mappability tracks were created using GEM library (gem-mappability)^62^. Two published pipelines, CORTEX^63^ and BRESEQ^64^, were additionally used to detect SNPs and structural variants but no additional variants were found. Finally, assembly *de novo* of the unmapped reads was also performed using Velvet^65^ to find putative novel sequences out of the reference genome. Any mutations present in the ancestral clones were excluded, leaving only mutations which accumulated throughout the experiment.

## Additional information

**Accession codes**: The genome sequences generated in this work have been deposited in the European Nucleotide Archive database under the accession code PRJEB7066.

**How to cite this article**: San Millan, A. *et al.* Positive selection and compensatory adaptation interact to stabilize non-transmissible plasmids. *Nat. Commun.* 5:5208 doi: 10.1038/ncomms6208 (2014).

## Supplementary Material

Supplementary Information Supplementary Figures 1-9, Supplementary Tables 1-3, Supplementary Methods and Supplementary References

## Figures and Tables

**Table 1:** Parameters used in the numerical simulations of the model.

**Parameter**	**Description**	**Value**	**Source**
ρ_*_	Resource conversion coefficient (*B*, *B*_p_, *C*, *C*_p_)	(1.232, 0.925, 0.916, 1.198) × 10^9^	OD_600_ data (MCMC)
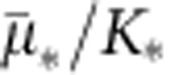	Specific affinity (*B*, *B*_p_, *C*, *C*_p_)	(6.305, 8.162, 10.704, 6.627) × 10^−10^	OD_600_ data (MCMC)
σ_*_	Segregation rate	*σ*_b_=0.00479, *σ*_c_=0.01223	qPCR data
γ_*_	Antibiotic killing rate	*γ*=0.1, *γ*_p_=18 × 10^−4^	MIC data
α_*_	Antibiotic degradation rate	*α*=10 × 10^−8^, *α*_p_=10 × 10^−7^	Drug degradation data
*ε*	Rate of point mutation	10 × 10^−7^	
*δ*	Dilution parameter	0.1% of volume	

MCMC, Markov-chain Monte Carlo; MIC, minimal inhibitory concentration; qPCR, quantitative PCR.
