# Supplementary material for: Positive selection and compensatory adaptation interact to stabilize non-transmissible plasmids
Source: Nat Commun. 2014 Oct 10;5:5208. doi: 10.1038/ncomms6208 (PMC4208098; doi:10.1038/ncomms6208)
Supplement: Supplementary Information — Supplementary Figures 1-9, Supplementary Tables 1-3, Supplementary Methods and Supplementary References [file ncomms6208-s1.pdf]

Supplementary Figure 1

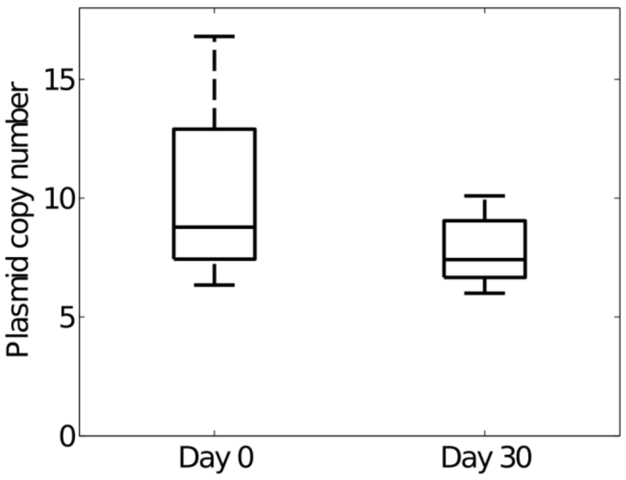

Copy number of pNUK73.

Number of pNUK73 copies per cell in the ancestral (day 0, n=5) and compensated (day 30, n=6) PA01/pNUK73 clones. Copy numbers were determined by qPCR. The line inside the box marks the median. The upper and lower hinges correspond to the 25th and 75th percentiles. The upper and lower whiskers extend to the highest and lowest values.

Supplementary Figure 2

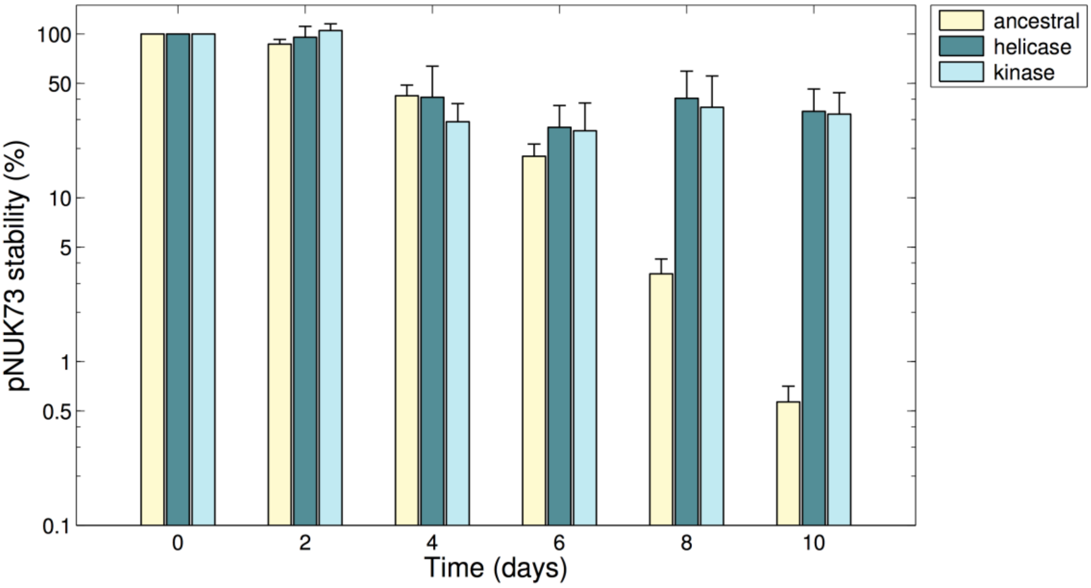

Stability of pNUK73 in the compensated clones.

This figure shows the relative proportion (average ± s.d.) of plasmids-bearing bacteria (logarithmic scale) over 10 days of serial passage (approximately 100 generations) for three populations of the parental PAO1/pNUK73 strain and six populations of two different clones showing compensatory mutations in the putative helicase gene (PA1372), 30+_1_2 or in one of the two contiguous putative kinases genes (PA4673.15), 30+_2_2. In Table S1 we provide with a detailed description of the mutations of each clone.

Supplementary Figure 3

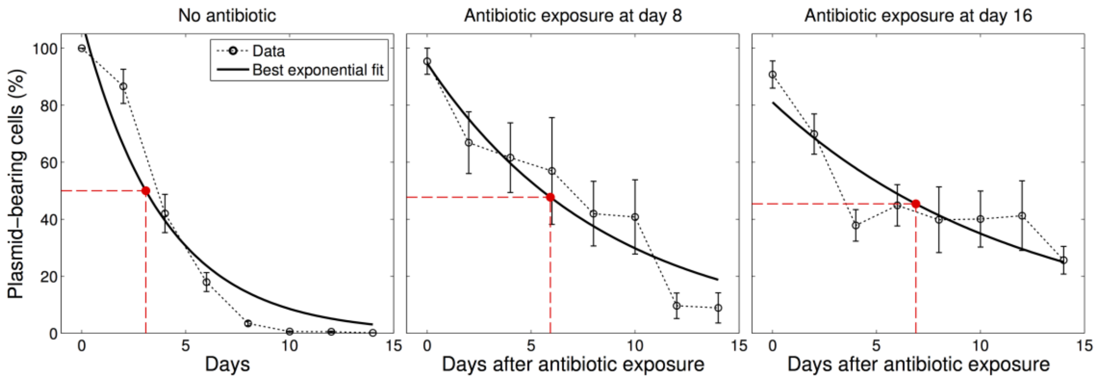

Increase in plasmid half-life after antibiotic exposure.

Plasmid half-life computed by interpolating an exponential fit to the observed frequency of plasmid bearing clones when (A) no antibiotic was used (plasmid half-life: 3.08 days), B) drug was used at day 8 (half-life: 5.93 days), and (C) drug was used at day 16 (half-life: 6.89 days). Note how, as predicted by the mathematical model, delays in antibiotic exposure have the effect of stabilizing the plasmid and increasing the plasmid's half-life.

Supplementary Figure 4

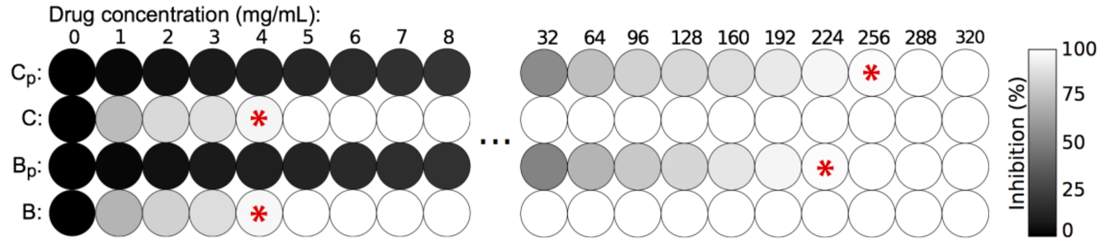

Antibiotic suppression parameters obtained from dose-response experiments.

Bacterial densities at the end of a 24 hour model simulation under increasing concentrations of antibiotic are represented with the filled circles: black represents no inhibition and white no growth detected. The circles highlighted with the red star correspond to the minimum inhibitory concentration where no visible bacterial growth was observed experimentally.

Supplementary Figure 5

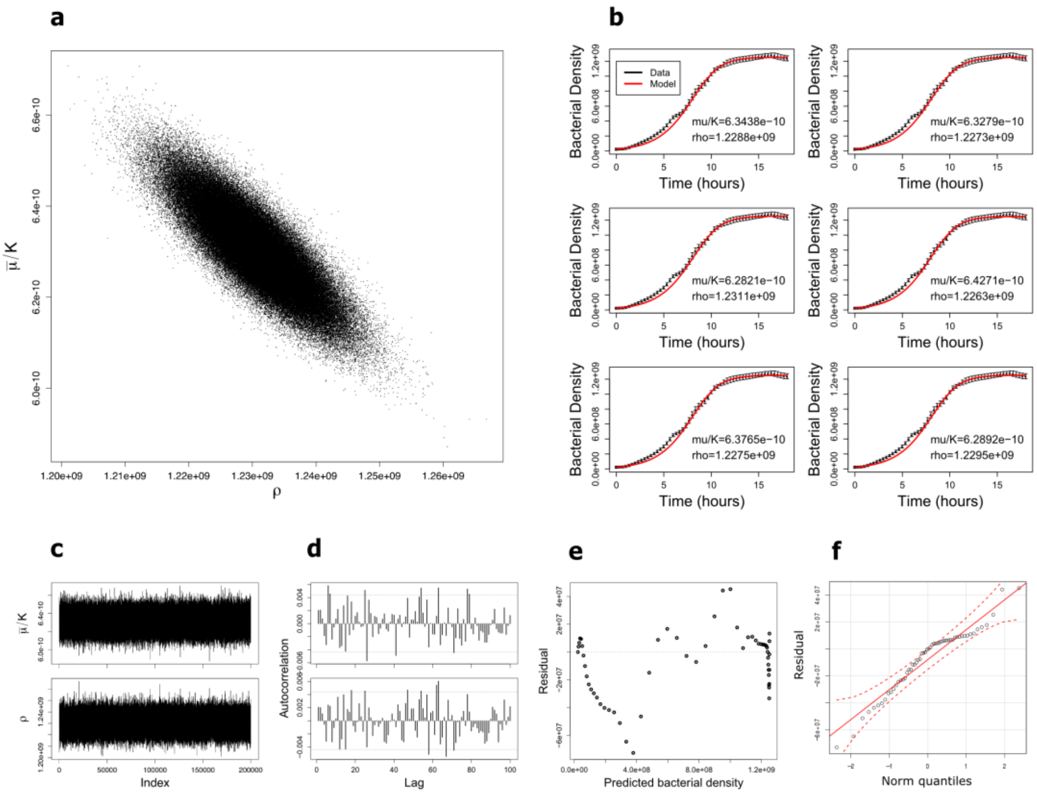

MCMC diagnostics: parental strain, plasmid-free.

a) 2-dimensional posterior distribution. b) Comparison between data (dots with standard error) and model simulations (red solid line) using parameters selected randomly from the posterior distribution. c) Traces of chains for parameters $\bar{\mu}/K$ (above) and ρ (below). d) Autocorrelation functions for both parameters. e) Residual plot for the time series data. f) Q-Q plot for the residual.

Supplementary Figure 6

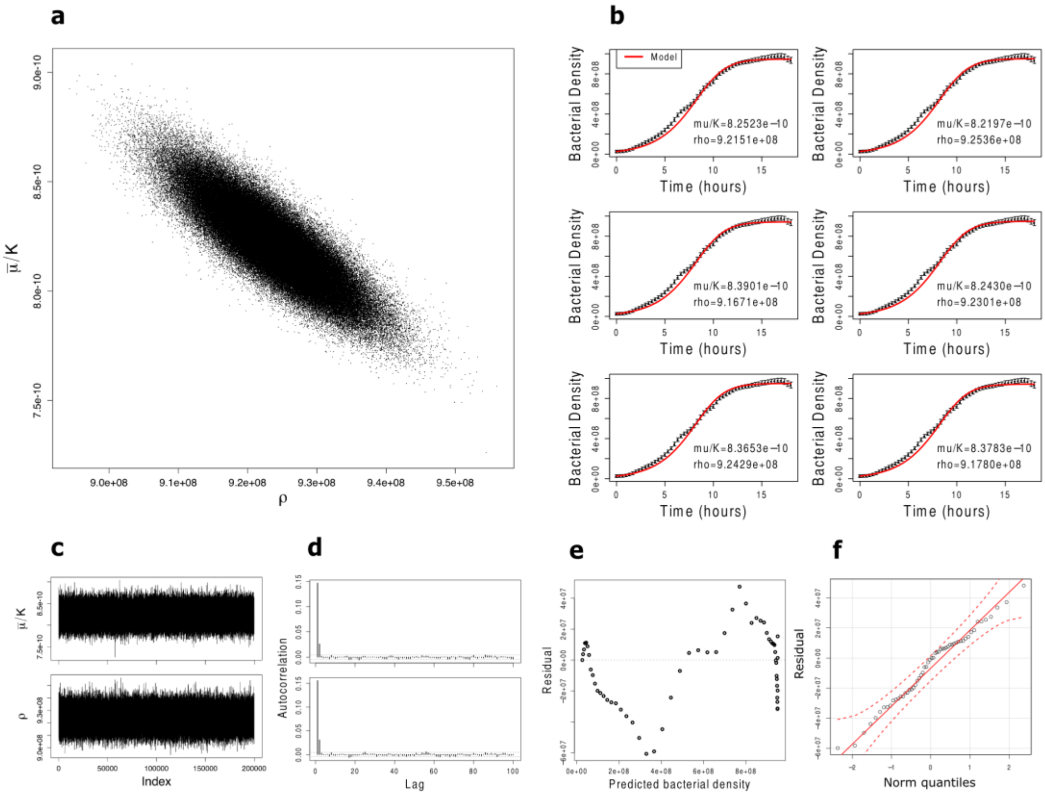

MCMC diagnostics: parental strain, plasmid-bearing.

a) 2-dimensional posterior distribution. b) Comparison between data (dots with standard error) and model simulations (red solid line) using parameters selected randomly from the posterior distribution. c) Traces of chains for parameters $\bar{\mu}/K$ (above) and ρ (below). d) Autocorrelation functions for both parameters. e) Residual plot for the time series data. f) Q-Q plot for the residual.

Supplementary Figure 7

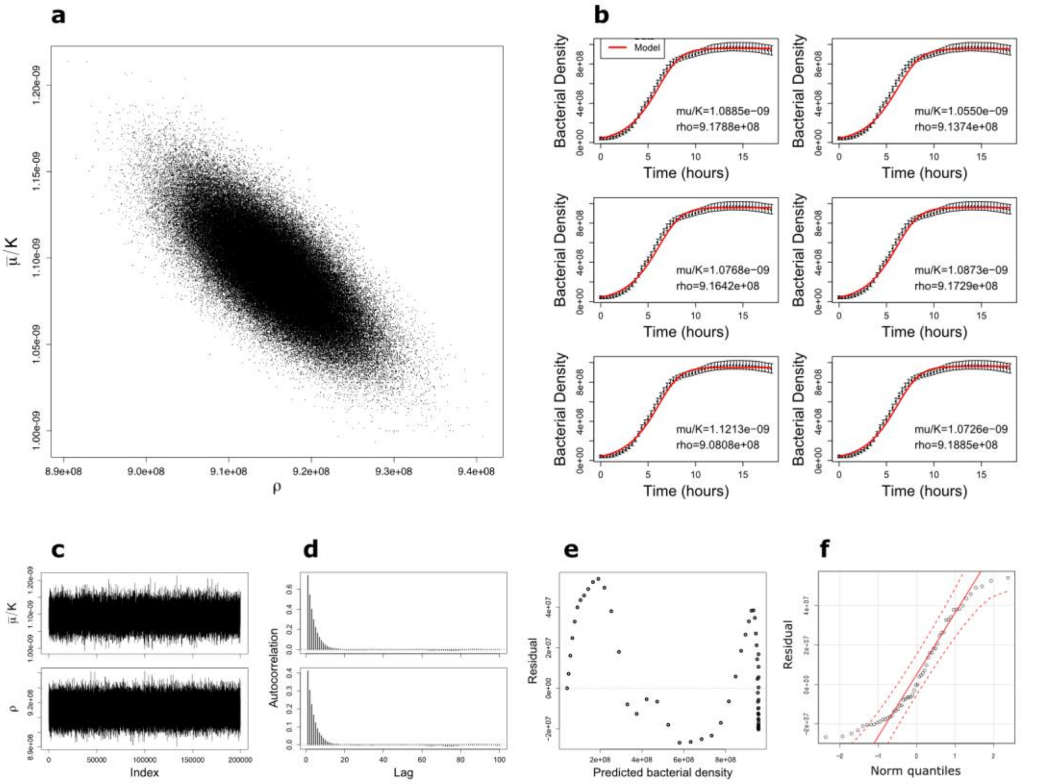

MCMC diagnostics: compensated strain, plasmid-free.

a) 2-dimensional posterior distribution. b) Comparison between data (dots with standard error) and model simulations (red solid line) using parameters selected randomly from the posterior distribution. c) Traces of chains for parameters $\bar{\mu}/K$ (above) and ρ (below). d) Autocorrelation functions for both parameters. e) Residual plot for the time series data. f) Q-Q plot for the residual.

Supplementary Figure 8

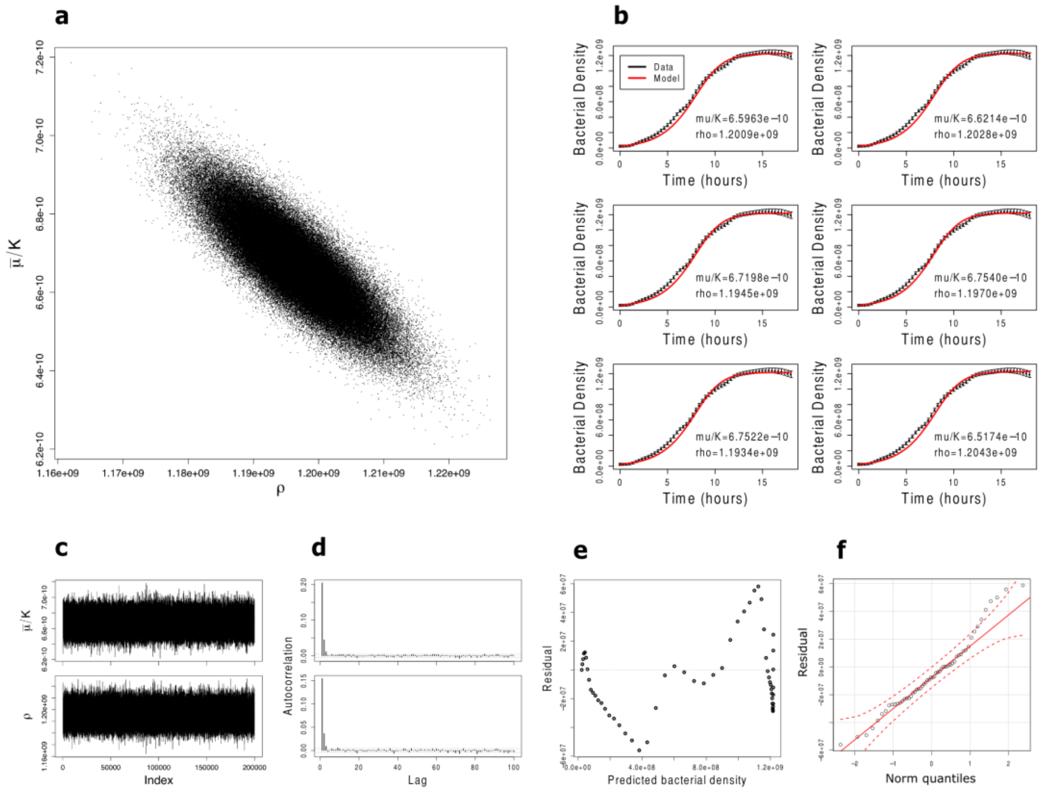

MCMC diagnostics: compensated strain, plasmid-bearing.

a) 2-dimensional posterior distribution. b) Comparison between data (dots with standard error) and model simulations (red solid line) using parameters selected randomly from the posterior distribution. c) Traces of chains for parameters $\bar{\mu}/K$ (above) and ρ (below). d) Autocorrelation functions for both parameters. e) Residual plot for the time series data. f) Q-Q plot for the residual.

Supplementary Figure 9

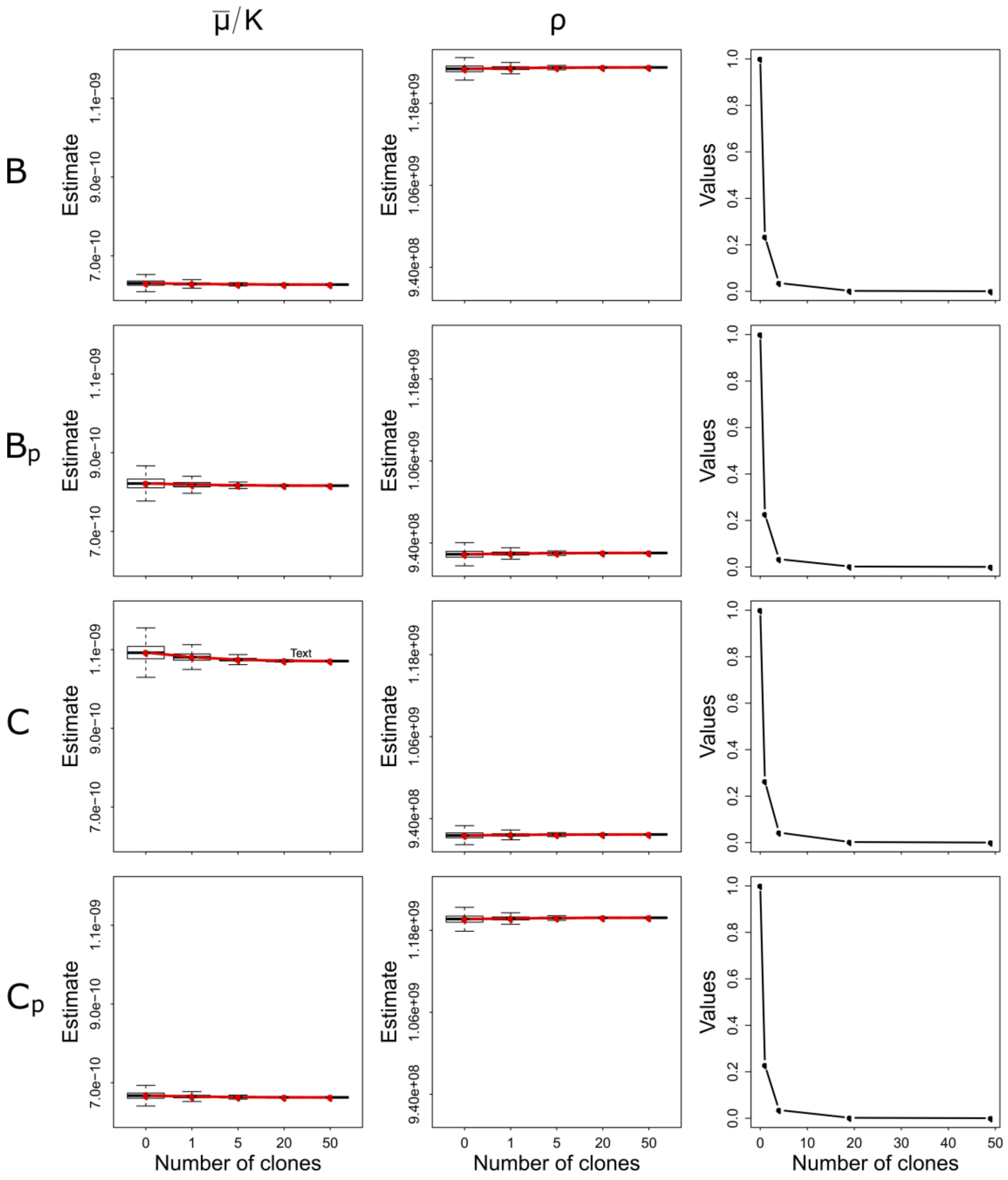

Data cloning convergence diagnostics plots.

Each row corresponds to a different bacterial type, from top to bottom: B, B_p, C, C_p. In all cases we used a uniform prior and kept 5000 posterior MCMC draws after discarding burn-in. Left and middle columns show parameter estimates as a function of the number of clones for $\bar{\mu}/K$ and ρ respectively. Note how the mean values of the posterior distribution (red lines) are converging towards the maximum likelihood estimates and the standard errors (vertical lines) are getting smaller as the number of clones increases. Plots in the right column illustrate how the standardized maximum eigenvalue also converges to zero as the number of clones increases, indicating estimability of the parameters.

Supplementary Table 1. Description of the mutations in the clones analyzed in this work.

Mutations by gene

Gene id	Product name	Position	Mutation	Effect	Mutated clones
PA1372	putative helicase	1489043	SNP C-->T	Non-synonymous	30+_2_1
PA1372	putative helicase	1487154	SNP C -->T	Non-synonymous	8S+_3_1
PA1372	putative helicase	1487965	Deletion 1nt (T)	Frameshift	30+_1_2
PA1372	putative helicase	1488678	HZ SNP C(74%)-->T(26%)	Non-synonymous	30S+_1_2
PA1372	putative helicase	1487590	Deletion 1nt (G)	Frameshift	8S+_3_2
PA1372	putative helicase	1486998	Deletion 1223 nt	frameshift	8S+_1_2
PA1695	PscP (translocation protein in type III secretion)	1844903	HZ Deletion 6 nt (48%)	Codon change + codon deletion	30S-_3_1
PA2020	MexZ (probable transcriptional regulator)	2212925	Deletion 72 nt	Codon deletion	8S+_2_1
PA2294	probable ATP-binding component of ABC transporter	2524944	SNP C-->T	Non-synonymous	8S+_3_3
intergenic	intergenic	2558272	Deletion 526 nt	intergenic	8S+_1_3
PA2402	probable non-ribosomal peptide synthetase	2686632	HZ Deletion 11 nt (21%)	Frameshift	30S+_1_2
PA2526	MuxC (multidrug efflux pump)	2848293	SNP A-->T	Non-synonymous	8S+_3_3
PA2839	putative dioxygenase	3193349	SNP G-->A	Non-synonymous	30-_3_1
PA3264	Probable transporter	3652530	HZ SNP G(83%)-->A(17%)	Synonymous	30-_2_3
PA3350	flagellar basal body P-ring biosynthesis protein FlgA	3762613	Deletion 55 nt	Frameshift	30S+_2_2

PA3539	conserved hypothetical protein	3961617	SNP G-->A	Synonymous	30-_2_2
PA3703	WspF (methylesterase)	4145103	SNP G-->A	Stop codon gained	30S+_1_1, 30S+_3_1, 30S+_3_2, 30-_1_1, 30S-_1_2, 30S-_1_3, 30S-_3_1
PA3703	WspF (methylesterase)	4145834	Insertion 46 nt	Codon insertion	8S+_1_1, 8S+_2_1, 8S+_3_1, 30-_1_2, 30-_2_1, 30-_2_2, 30-_2_3, 30-_3_1, 30-_3_2, 30S-_1_1, 30S-_2_1, 30S-_2_2, 30S-_2_3, 30S-_3_3
PA3703	WspF (methylesterase)	4145836	Deletion 92 nt	Frameshift	30S+_2_1
PA3703	WspF (methylesterase)	4145463	Deletion 98 nt	Frameshift	30S-_3_2
PA4673.15	putative protein kinase	5252149	Deletion 1 nt	Frameshift	30+_2_2, 30+_3_1, 30S+_2_1
PA4673.15	putative protein kinase	5252204	SNP G-->A	Non-synonymous	30S+_3_1, 30S+_3_2
PA4673.15	putative protein kinase	5252585	Deletion 1 nt	Frameshift	30+_3_2, 30S+_2_2
PA4673.15	putative protein kinase	5252617	SNP G-->T	Stop codon gained	30S+_1_1
PA4673.16	putative protein kinase	5252983	SNP G-->A	Non-synonymous	30+_1_1
PA4673.16	putative protein kinase	5253760	HZ Insertion 1 nt (66%)	Frameshift	30S+_1_2, 8S+_3_3
intergenic	intergenic	5269310	SNP T-->C	intergenic	8S+_1_3
PA5017	putative diguanylate cyclase	5642825	SNP A-->C	Non-synonymous	30-_1_3
PA5017	putative diguanylate cyclase	5642856	Deletion 6nt	Codon change + codon deletion	8S+_1_3

Mutations by clone

Mutated clones	Gene id	Product name	Position	Mutation	Effect
**Clones evolved with no antibiotics**					
**30 days, PAO1/pNUK73**					

30+_1_1	PA4673.16	putative protein kinase	5252983	SNP G-->A	Non-synonymous
30+_1_2	PA1372	putative helicase	1487965	Deletion 1nt (T)	Frameshift
30+_2_1	PA1372	putative helicase	1489043	SNP C-->T	Non-synonymous
30+_2_2	PA4673.15	putative protein kinase	5252149	Deletion 1 nt	Frameshift
30+_3_1	PA4673.15	putative protein kinase	5252149	Deletion 1 nt	Frameshift
30+_3_2	PA4673.15	putative protein kinase	5252585	Deletion 1 nt	Frameshift
**30 days, PAO1 (no pNUK73)**					
30-_1_1	PA3703	WspF (methylesterase)	4145103	SNP G-->A	Stop codon gained
30-_1_2	PA3703	WspF (methylesterase)	4145834	Insertion 46 nt	Codon insertion
30-_1_3	PA5017	putative diguanylate cyclase	5642825	SNP A-->C	Non-synonymous
30-_2_1	PA3703	WspF (methylesterase)	4145834	Insertion 46 nt	Codon insertion
30-_2_2	PA3539	conserved hypothetical protein	3961617	SNP G-->A	Synonymous
	PA3703	WspF (methylesterase)	4145834	Insertion 46 nt	Codon insertion
30-_2_3	PA3264	Probable transporter	3652530	HZ SNP G(83%)-->A(17%)	Synonymous
	PA3703	WspF (methylesterase)	4145834	Insertion 46 nt	Codon insertion
30-_3_1	PA2839	putative dioxygenase	3193349	SNP G-->A	Non-synonymous
	PA3703	WspF (methylesterase)	4145834	Insertion 46 nt	Codon insertion
30-_3_2	PA3703	WspF (methylesterase)	4145834	Insertion 46 nt	Codon insertion
30-_3_3					
**Clones evolved with antibiotics (day 8)**					
**After treatment, PAO1/pNUK73**					
8S+_1_1	PA3703	WspF (methylesterase)	4145834	Insertion 46 nt	Codon insertion
8S+_1_2	PA1372	putative helicase	1486998	Deletion 1223 nt	frameshift

8S+_1_3	intergenic	intergenic	2558272	Deletion 526 nt	intergenic
	intergenic	intergenic	5269310	SNP T-->C	intergenic
	PA5017	putative diguanylate cyclase	5642856	Deletion 6nt	Codon change + codon deletion
8S+_2_1	PA2020	MexZ (probable transcriptional regulator)	2212925	Deletion 72 nt	Codon deletion
	PA3703	WspF (methylesterase)	4145834	Insertion 46 nt	Codon insertion
8S+_2_2		No mutations			
8S+_2_3		No mutations			
8S+_3_1	PA1372	putative helicase	1487154	SNP C -->T	Non-synonymous
	PA3703	WspF (methylesterase)	4145834	Insertion 46 nt	Codon insertion
8S+_3_2	PA1372	putative helicase	1487590	Deletion 1nt (G)	Frameshift
8S+_3_3	PA2294	probable ATP-binding component of ABC transporter	2524944	SNP C-->T	Non-synonymous
	PA2526	MuxC (multidrug efflux pump)	2848293	SNP A-->T	Non-synonymous
	PA4673.16	putative protein kinase	5253760	Insertion 1 nt	Frameshift
**30 days, PAO1/pNUK73**					
30S+_1_1	PA4673.15	putative protein kinase	5252617	SNP G-->T	Stop codon gained
	PA3703	WspF (methylesterase)	4145103	SNP G-->A	Stop codon gained
30S+_1_2	PA1372	putative helicase	1488678	HZ SNP C(74%)-->T(26%)	Non-synonymous
	PA2402	probable non-ribosomal peptide synthetase	2686632	HZ Deletion 11 nt (21%)	Frameshift
	PA4673.16	putative protein kinase	5253760	HZ Insertion 1 nt (66%)	Frameshift
30S+_2_1	PA3703	WspF (methylesterase)	4145836	Deletion 92 nt	Frameshift

	PA4673.15	putative protein kinase	5252149	Deletion 1 nt	Frameshift
30S+_2_2	PA4673.15	putative protein kinase	5252585	Deletion 1 nt	Frameshift
	PA3350	flagellar basal body P-ring biosynthesis protein FlgA	3762613	Deletion 55 nt	Frameshift
30S+_3_1	PA4673.15	putative protein kinase	5252204	SNP G-->A	Non-synonymous
	PA3703	WspF (methylesterase)	4145103	SNP G-->A	Stop codon gained
30S+_3_2	PA4673.15	putative protein kinase	5252204	SNP G-->A	Non-synonymous
	PA3703	WspF (methylesterase)	4145103	SNP G-->A	Stop codon gained
**30 days, PAO1 (no pNUK73)**					
30S-_1_1	PA3703	WspF (methylesterase)	4145834	Insertion 46 nt	Codon insertion
30S-_1_2	PA3703	WspF (methylesterase)	4145103	SNP G-->A	Stop codon gained
30S-_1_3	PA3703	WspF (methylesterase)	4145103	SNP G-->A	Stop codon gained
30S-_2_1	PA3703	WspF (methylesterase)	4145834	Insertion 46 nt	Codon insertion
30S-_2_2	PA3703	WspF (methylesterase)	4145834	Insertion 46 nt	Codon insertion
30S-_2_3	PA3703	WspF (methylesterase)	4145834	Insertion 46 nt	Codon insertion
30S-_3_1	PA1695	PscP (translocation protein in type III secretion)	1844903	HZ Deletion 6 nt (48%)	Codon change + codon deletion
	PA3703	WspF (methylesterase)	4145103	SNP G-->A	Stop codon gained
30S-_3_2	PA3703	WspF (methylesterase)	4145463	Deletion 98 nt	Frameshift
30S-_3_3	PA3703	WspF (methylesterase)	4145834	Insertion 46 nt	Codon insertion

Examples of clone nomenclature of clones in this work:

30S+_3_2; 30, clone isolated at the end of the experiment. S, it has been subjected to one step of selection pressure from day 8 to 9. +, the clone carries pNUK73. _3, population 3. _2, clone 2 within the population 3.
30-_1_1: 30, clone isolated at the end of the experiment. -, the clone is plasmid-free. _1, population 1. _1, clone 1 within population 1.
8S+_2_2: 8, clone isolated after antibiotic treatment at day 8 (technically it was isolated after day 9). +, the clone carries pNUK73. _2, population 2. _2, clone 2 within the population 2.

Supplementary Table 2. Clones used to determine growth kinetic parameters.

Strain	*Description*	*Isolates*
B_p	Parental, plasmid-bearing	*PAO1/pNUK73*
B	Parental, plasmid-free	*PAO1*
C_p	Compensated, plasmid-bearing	30+_1_1, 30+_1_2, 30+_2_1, 30+_2_2, 30+_3_1, 30+_3_3
C	Compensated, plasmid-free	30__1_1, 30__1_2, 30__1_3, 30__2_1, 30__2_2, 30__2_3, 30__3_1,30__3_2, 30__3_2

Bacterial isolates used to determine growth kinetic parameters for each bacterial type defined in the evolutionary model. Parameter estimates were obtained by fitting growth curves of each isolate acquired by measuring optical densities at 600*nm* in a 96-well plate every 20 minutes using the same environmental conditions as the competition experiment (with 4 replicates, except for the parental strain that was replicated 3 times).

Supplementary Table 3. Estimated parameter values for different prior distributions.

B	*Priors*	ρ (×10⁹)	$\bar{\mu}/K$ (×10⁻¹⁰)
	MCMC 1	1.2305 [1.218, 1.243]	6.3055 [6.143, 6.473]
	MCMC 2	1.2283 [1.216, 1.241]	6.3363 [6.173, 6.509]
	MCMC 3	1.2283 [1.216, 1.241]	6.3365 [6.173, 6.506]
	MCMC 4	1.2306 [1.218, 1.243]	6.3039 [6.140, 6.472]
B_p	*Priors*	ρ ($\times 10^{8}$)	$\bar{\mu}/K$ ($\times 10^{-10}$)
	MCMC 1	9.2355 [9.107, 9.365]	8.2197 [7.896, 8.556]
	MCMC 2	9.2028 [9.072, 9.331]	8.3100 [7.983, 8.661]
	MCMC 3	9.2025 [9.071, 9.331]	8.3113 [7.983, 8.662]
	MCMC 4	9.2372 [9.109, 9.367]	8.2150 [7.892, 8.551]
C	*Priors*	ρ ($\times 10^{8}$)	$\bar{\mu}/K$ ($\times 10^{-10}$)
	MCMC 1	9.1543 [9.051, 9.257]	1.0924 [1.048, 1.140]
	MCMC 2	9.1316 [9.026, 9.232]	1.1043 [1.059, 1.153]
	MCMC 3	9.1317 [9.027, 9.234]	1.1043 [1.059, 1.150]
	MCMC 4	9.1555 [9.052, 9.259]	1.0916 [1.047, 1.138]
C_p	*Priors*	ρ ($\times 10^{9}$)	$\bar{\mu}/K$ ($\times 10^{-10}$)
	MCMC 1	1.1963 [1.183, 1.209]	6.6734 [6.480, 6.873]
	MCMC 2	1.1937 [1.181, 1.206]	6.7144 [6.521, 6.918]
	MCMC 3	1.1938 [1.180, 1.207]	6.7141 [6.522, 6.919]
	MCMC 4	1.1963 [1.183, 1.209]	6.6724 [6.480, 6.871]

Parameter estimates [95% confidence intervals] obtained using the MCMC algorithm with 2×10^{7} iterations kept after burn-in. MCMC 1: priors used were uniform ($0, 1 \times 10^{-8}$) for $\bar{\mu}/K$ and uniform($0, 1 \times 10^{11}$) for ρ. MCMC 2: uses lognormal priors with mean 0 and standard deviation equal to 1 for both parameters. MCMC 3: uses a beta(1,1) prior for $\bar{\mu}/K$ and a uniform($0, 1 \times 10^{11}$) prior for ρ. MCMC 4: uses a gamma(0.001,0.001) prior for $\bar{\mu}/K$ and uniform($0, 1 \times 10^{11}$) prior for ρ.

Supplementary Methods.

Model parametrization

First, we will determine growth kinetic parameters for different bacterial strains isolated from the plasmid stability experiment (see Supplementary Table 2). Then, once we have obtained estimates for the parameters that characterize the growth of each strain in a single season, we will simulate a serial transfer experiment using the evolutionary model described in the manuscript. The goal of this approach is to predict the ecological dynamics of a competition experiment based on observations of how each strain grows independently. The remaining parameters of the model will be determined using values obtained from the literature (rate of point mutation) or using other experimental data (antibiotic susceptibility, drug degradation and rate of plasmid segregation).

Let us begin by denoting the bacterial density at time t with the variable $x(t)$ and with $R(t)$ the concentration of environmental resource. Then bacterial growth in a homogeneous environment with resource limitation can be modelled with the following equations:

$$(1) \qquad \frac{dR}{dt} = -\left(\frac{\bar{\mu}\, R(t)}{K + R(t)}\right) x,$$

$$(2) \qquad \frac{dx}{dt} = \rho\left(\frac{\bar{\mu}\ R(t)}{K + R(t)}\right)$$

with initial conditions (R_0, x_0). Here ρ denotes a resource conversion coefficient, $\bar{\mu}$ represents the maximum growth rate and K the cell's affinity for the resource. This model is indeed very simple, but it has been extensively used before to describe bacterial growth in batch reactor experimental systems [8, 17] and will be the core of the evolutionary model used in the main text to study plasmid dynamics.

Despite the advantages of using mechanistic models, this approach also requires some caveats. For instance, it is known that ecological and biophysical models can present identifiability problems [9,13,5] and as a consequence parametrization algorithms may fail to converge or to provide reliable estimates for all parameters. In particular, microbial growth models that contain Michaelis-Menten type nonlinearities, like the one described by equations (1-2) can be nonidentifiable due to $\bar{\mu}$ and K being highly correlated [6,8]. It has been reported that the system can be structurally identifiable if we can measure the initial concentration of limiting resource [3], but not if we only measure bacterial optical density, which is the case in our experimental setup. To overcome this limitation, it has been proposed that instead of trying to estimate values for $\bar{\mu}$ and K, the $\bar{\mu}/K$ ratio should be used to assess competitive fitness between different strains [4,7], a quantity referred to in the literature as *specific affinity* [8].

It is important to highlight that the objective of the mathematical model presented in this paper is not to describe the internal biological processes of the cell or to assign biological interpretation to the estimated growth kinetic parameters, but to quantify the fitness cost associated with plasmid-bearing, as well as the fitness advantage of acquiring a compensatory mutation relative to the parental strain. Therefore we will use the simple Monod model described by Supplementary Equations (1-2) as an empirical metabolic

model whereby the specific affinity ($\bar{\mu}/K$) and the resource conversion coefficient (ρ) will provide us with a measure of the relative fitness of different strains. Both parameters were jointly estimated by fitting the Monod model to growth curve data (optical density measurements) assuming normally distributed errors using a Metropolis-Hastings Markov-chain Monte Carlo (MCMC) method implemented in R, a free open-source statistical package [14], with scripts available in a public repository [16].

The estimated parameter values for different prior distributions are summarized in Supplementary Table 3. In all cases, we used 2.5×10^7 iterations with a burn-in period of 5×10^6 and a thinning of 100 iterations. In order to adjust the acceptance probabilities for the proposed updates we used an adaptive proposal variance method as a first step of the MCMC algorithm. During this period no samples were stored in the chain until the acceptance ratio reached a target value, then we fixed the proposal variance and started storing the MCMC iterations. Convergence of the Markov chains was assessed by visual inspection.

The resulting posterior distributions, as well as the diagnostic plots of the MCMC algorithm for each one of the bacterial strains are presented in Figures S5-S8. Note how, although the MCMC chains appear to have converged and the estimated parameters provide a good fit to the data capturing the essential qualitative features, this simple model systematically underestimates the bacterial density during the first few hours of exponential phase. This could be a consequence of the rich media used in the experiments (LB broth) containing multiple limiting substrates [15]. While we could, of course, pose more complex models to capture these features [1, 12], for the questions we address in this paper we consider the simpler evolutionary model to be more appropriate [18].

Furthermore, in order to assess the estimability of the parameters of our model and to obtain maximum likelihood estimates for each parameter, we implemented a data-cloning method as described in [10,11]. This method is based on a hypothetical situation whereby an individual performs simultaneously *k* independent experiments and by coincidence produces the same results. This is, of course, an unrealistic scenario, but we can simulate it by generating *k* clones of the original data and using a Metropolis-Hastings MCMC method modified to consider a new likelihood function given by the original function raised to the *k*-th power. Lele and co-authors demonstrated in [10,11] that as the number of clones increases, the marginal posterior distribution converges to a multivariate normal distribution with mean equal to the maximum likelihood estimate with approximate variance corresponding to *k* times the posterior variance. Supplementary Fig. 9 illustrates the results obtained after applying the data cloning algorithm to our model and optical density data.

Finally, we used the ANOVA test proposed in [2] to confirm that the parameters are estimable and that the estimates obtained using the MCMC algorithm are reliable. The objective of this test is to reject the null hypothesis that there are significant differences in estimates when changing the number of clones or when considering different prior distributions. For instance, the plasmid-bearing parental strain with $k = \{1000, 2000\}$ clones, four different prior distributions (described in Supplementary Table 3) and 40000 posterior samples kept after burn-in produced a *p*-value for cloning effect of 0.7012 for ρ and 0.4025 for $\bar{\mu}/K$, and a *p*-value for prior effect of 0.8152 and 0.9106 respectively (analogous *p*-values were also obtained for the other bacterial types). These values indicate that there are no significant cloning or prior effects and as a consequence we conclude that the MCMC chains have converged to the maximum likelihood estimates. We will use the

obtained parameter values (summarized in Table 1) to characterize the relative fitness of each strain in the plasmid dynamics model presented in the main text of this paper.

Supplementary References

[1] H Beyenal, SN Chen, and Z Lewandowski. The double substrate growth kinetics of pseudomonas aeruginosa. *Enzyme Microbial Technol*, 32(1):92–98, 2003.

[2] D Campbell and SR Lele. An ANOVA test for parameter estimability using data cloning with application to statistical inference for dynamic systems. *Comput Stat Data An*, 70(2014):257–267, February 2014.

[3] MJ Chappell and KR Godfrey. Structural identifiability of the parameters of a nonlinear batch reactor model. *Math Biosci*, 251:241–251, 1992.

[4] FP Healey. Slope of the monod equation as an indicator of advantage in nutrient competition. *Microb Ecol*, 5(4):281–286, 1980.

[5] KE Hines, TR Middendorf, and RW Aldrich. Determination of parameter identifiability in nonlinear biophysical models: A bayesian approach. *J Gen Physiol*, 143(3):401–416, 2014.

[6] A Holmberg. On the practical identifiability of microbial growth models incorporating Michaelis-Menten type nonlinearities. *Math Biosci*, 43:23–43, 1982.

[7] D-J Kim, J-W Choi, N-C Choi, B Mahendran, and C-E Lee. Modeling of growth kinetics for pseudomonas spp. during benzene degradation. *Appl Microbiol Biotechnol*, 69(4):456–462, 2005.

[8] K Kovárová-Kovar and T Egli. Growth kinetics of suspended microbial cells: from single-substrate-controlled growth to mixed-substrate kinetics. *Microbiology and molecular biology reviews*, 62(3):646–666, 1998.

[9] SR Lele and Dennis B. Bayesian methods for hierarchical models: are ecologists making a faustian bargain. *Ecol Appl*, 19(3):581–584, 2009.

[10] SR Lele, B Dennis, and F Lutscher. Data cloning: easy maximum likelihood estimation for complex ecological models using Bayesian Markov chain Monte Carlo methods. *Ecol Lett*, 10(7):551–63, July 2007.

[11] SR Lele, K Nadeem, and B Schmuland. Estimability and Likelihood Inference for Generalized Linear Mixed Models Using Data Cloning. *J Am Statist Assoc*, 105(492):1617–1625, December 2010.

[12] U Lendenmann, M Snozzi, and T Egli. Growth kinetics of escherichia coli with galactose and several other sugars in carbon-limited chemostat culture. *Can J Microbiol*, 46(1):72–80, 1999.

[13] JM Ponciano, ML Taper, B Dennis, and SR Lele. Hierarchical models in ecology: confidence intervals, hypothesis testing, and model selection using data cloning. *Ecology*, 90(2):356–362, 2009.

[14] R Core Team. *R: A Language and Environment for Statistical Computing*. R Foundation for Statistical Computing, Vienna, Austria, 2013. ISBN 3-900051-07-0.

[15] G Sezonov, D Joseleau-Petit, and R D'Ari. Escherichia coli physiology in Luria-Bertani broth. *J Bacteriol*, 189(23):8746–9, December 2007.

[16] A San Millan, R Peña-Miller, M Toll-Riera, Z Halbert, A McLean, BS Cooper, RC MacLean. A Metropolis-Hastings MCMC implementation used to fit a bacterial growth curve. figshare. http://dx.doi.org/10.6084/m9.figshare.1127995
Retrieved 22:35, Aug 05, 2014 (GMT)

[17] R Peña-Miller, A Fuentes-Hernandez, C Reding, I Gudelj and RE Beardmore. Testing the optimality properties of a dual antibiotic treatment in a two-locus, two-allele model. J Roy Soc Interface, 11(96), 20131035, 2014.
[18] A Gelman and CR Shalizi. Philosophy and the practice of Bayesian statistics. Brit J Math Stat, 66(1), 8–38, 2013.
